# PROTOCOL: Effects of interventions to improve access to financial services for micro‐, small‐ and medium‐sized enterprises in low‐ and middle‐income countries: An evidence and gap map

**DOI:** 10.1002/cl2.1341

**Published:** 2023-07-05

**Authors:** Nina Ashley Dela Cruz, Alyssa Cyrielle B. Villanueva, Lovely Ann Tolin, Sabrina Disse, Robert Lensink, Howard White

**Affiliations:** ^1^ Campbell Collaboration Las Pinas City Philippines; ^2^ Campbell Collaboration Meycauayan City Bulacan Philippines; ^3^ Campbell Collaboration Quezon City Philippines; ^4^ Campbell Collaboration Cologne Germany; ^5^ University of Groningen Groningen The Netherlands; ^6^ Campbell Collaboration New Delhi India

## Abstract

**Background:**

Micro‐, small‐, and medium‐sized enterprises (MSMEs) account for the vast majority of firms in most economies, particularly in developing nations, and are key contributors to job creation and global economic development. However, the most significant impediment to MSME development in low‐ and middle‐income countries is a lack of access to both investment and working capital financing. Due to a lack of essential track record, appropriate collateral, and credit history, MSMEs are frequently denied business loans by traditional lending institutions. In addition, SMEs’ inability to access funding is hindered by institutional, structural, and non‐financial factors. To address this, both the public and private sectors employ indirect and direct finance interventions to help MSMEs in developing and emerging economies enhance and increase their financing needs. Given the importance of MSMEs in the economy, a comprehensive overview of and systematic synthesizing of the evidence of the effects of financial access interventions for MSMEs, capturing a wide variety of outcome variables, is useful.

**Objectives:**

The objective of this evidence and gap map (EGM) is to describe the existing evidence on the effects of various interventions dedicated to supporting and improving MSMEs’ access to credit, as well as the corresponding firm performance and/or welfare outcomes.

**Methods:**

An EGM is a systematic evidence product that displays the existing evidence relevant to a specific research question. An EGM's end product is a research article or report, but it can also be shared via an interactive map drawn as a matrix of included studies and their corresponding interventions and outcomes. Interventions in low‐ and middle‐income countries that target specific population subgroups are included on the map. The EGM considers five types of interventions: (i) strategy, legislation and regulatory; (ii) systems and institutions; (iii) facilitate access; (iv) lending instruments or financial products; and (v) demand‐side interventions. The map, on the other hand, covers outcome domains for policy environment, financial inclusion, firm performance, and welfare. Impact evaluations or systematic reviews of relevant interventions for a previously defined target population are included in the EGM. Studies using experimental or non‐experimental designs, as well as systematic reviews, are eligible. The EGM excludes before‐and‐after study designs with no suitable comparison group. Furthermore, the map excludes literature reviews, key informant interviews, focus group discussions, and descriptive analyses. Search strings were used to conduct electronic searches in databases. To ensure that the research team had identified a significant portion of relevant research works, the search strategy was supplemented with gray literature searches and systematic review citation tracking. We have compiled studies that are either completed or in progress. For practical reasons, studies are limited to papers written in English and are not restricted by publication date.

**Selection Criteria:**

We included studies that examined interventions to enhance MSMEs’ access to finance in low‐ and middle‐income countries targeting MSMEs including households, smallholder farmers and single person enterprise as well as financial institutions/agencies and their staff. The EGM considers five types of interventions that aim to: (i) deliver strategy, legislation, and regulatory aspects; (ii) systems and institutions that enable financing; (iii) facilitate access to finance; (iv) deliver different lending instruments or financial products, including traditional forms of microcredit; and (v) demand‐side interventions such as programs on financial literacy. The map includes outcome domains surrounding policy environment, financial inclusion, firm performance, and welfare. Eligible studies must be experimental, non‐experimental, or systematic reviews. In addition, the study designs must have a suitable comparison group before and after the implementation of interventions.

**Results:**

The EGM includes 413 studies. The majority of the studies (379 studies) analyzed microenterprises, such as households and smallholder farmers; 7 studies analyzed community groups; while 109 studies analyzed small and medium enterprises. There were 147 studies on interventions that targeted multiple firm sizes. Lending instruments/financial products are the most common intervention across all firm types. When it comes to the types of firms that receive the said financial intervention, the data is overwhelmingly in favor of microenterprises (278 studies), followed by systems and organizations (138 studies) that support better access to such financial products and services. Welfare outcomes have the most evidence out of all of the outcomes of interest, followed by firm performance and financial inclusion. Among all firm types, welfare outcomes are primarily targeted at microenterprises. With 59 studies, we can say that small businesses have a significantly large number of enterprise performance outcomes. of the 413 studies, 243 used non‐experimental or quasi‐experimental designs (mainly propensity score matching and instrumental variable approaches), 136 used experimental methods, and 34 were systematic reviews. 175 studies (43%) provided evidence from Sub‐Saharan Africa, 142 studies (35%) from South Asia, 86 studies (21%) from East Asia and the Pacific, 66 studies (16%) from Latin America and the Caribbean, 28 studies (7%), Europe and Central Asia, and 21 studies (5%) from the Middle East and North Africa. Most of the included evidence covers low‐income (26%) and lower‐middle income countries (66%), and to a lesser extent upper‐middle‐income countries (26%).

**Conclusion:**

This map depicts the existing evidence and gaps on the effects of interventions to enhance MSMEs’ access to financial services in low and middle‐income countries. Interventions directed at microenterprises with welfare outcomes have a significant number of research outcomes in the literature. SME evaluations have looked at firm performance, with less focus to employment and the welfare effects on owners and employees, including poverty reduction. Microcredit/loans have been the focus of a large number of research papers (238 studies), indicating the field's growing popularity. However, emerging financial interventions such as facilitating access to digital financial services are relatively under‐studied. Several studies also investigate rural or population in remote areas with 192 studies, 126 studies on poor and disadvantaged, and 114 papers on women. Most of the research is conducted in Sub‐Saharan Africa (175 studies) and South Asia (142 studies) so further research in other regions could be conducted to allow a more holistic understanding of the effects of financial inclusion interventions. Credit lines, supply chain finance, and trade financing, which are some of the ADB's financial tools have limited evidence. Future studies should look into strategy, law, and regulation interventions, as well as interventions targeted at SMEs, and examine policy and regulatory environment outcomes as well as welfare outcomes. Interventions on the demand side and their impact on the policy and regulatory environment, as well as facilitating access are relatively understudied.

## 1 BACKGROUND

### 1.1 The problem, condition, or issue

In most economies, particularly in developing countries, micro‐, small‐ and medium‐sized enterprises (MSMEs) make up the vast majority of businesses globally and are critical contributors to employment creation and global economic development. They account for around 90% of enterprises and two‐thirds of all jobs in the globe. Formal and informal MSMEs account for up to 50% of national income (GDP). To accommodate the rising global workforce, it is anticipated that 600 million jobs will be required by 2030, making MSME development a top priority for many governments around the world. As a result, MSME financing is important since MSMEs are at the heart of job creation (World Bank, n.d.).

However, the biggest hurdle to MSME development in low‐ and middle‐income countries is often described as a lack of access to finance for both investment and working capital (International Finance Corporation [IFC], 2017). The needs of MSMEs, especially micro‐enterprises, are often for small loans available on short notice, which the formal sector is often ill‐suited to provide. Instead, they use internal finances or cash from friends and family to start and run their businesses. MSMEs are also not considered eligible for business loans by traditional lending institutions, owing to a lack of necessary track record, acceptable collateral, and credit history. Furthermore, MSMEs may be discouraged by the tedious documentation process required for business loans (Organization for Economic Cooperation and Development [OECD], 2015).

According to estimates of the SME Finance Forum (2020), 131 million firms in developing nations, or 41% of formal MSMEs, have an annual financing need of $4.5 trillion (SME Finance Forum, 2020). East Asia and the Pacific has the highest share of the global finance gap (46%) followed by Latin America and the Caribbean (23%) and Europe and Central Asia (15%). More than half of all SMEs, both formal and informal, lack access to formal credit (World Bank, n.d.). Financing constraints are exacerbated for informal businesses, which are often small and contribute significantly to economic activity and employment despite being less productive than formal businesses. Unregistered businesses rely mostly on informal financing, which, while beneficial in terms of simplifying access to capital, is linked to lower enterprise development and higher illegality.

Even though the informal sector accounts for a large portion of unmet loan demand, many businesses remain informal due to a lack of incentives or capacity to formalize. It may take a long time to create an enabling environment for firms to formalize, as it not only requires the establishment of solid institutions, laws and regulations, infrastructure, and education, but it also necessarily requires the identification of business‐oriented incentives for firms, such as access to new market opportunities and access to financial and non‐financial services, making it a profitable decision for firms to register their business (IFC, 2013).

More generally, institutional, structural, and non‐financial factors all have a role in SMEs’ inability to obtain financing. Some of these factors are (i) lack of credit information when assessing borrowers’ creditworthiness; (ii) absence of alternatives to traditional collateral‐ based lending; (iii) ineffective debt recovery and poor business exit mechanisms; (iv) inaccessible financing instrument alternatives; and (v) lack of technological innovation in credit assessment practices (IFC, 2017). These internal and external challenges limit MSMEs’ development and potential to create jobs, contribute to GDP, and aid nations’ export competitiveness. The public sector has attempted to play several roles in bridging the financial access gap for SMEs. Financial inclusion programs, which aim to improve poor people's access to financial services, have been made available in low‐ and middle‐income countries to improve the households’. However, financial inclusion interventions appear to have unpredictable and possibly small effects, therefore the issue of inaccessible MSME financing persists (Duvendack & Mader, 2019). Therefore, both the public and private sectors use indirect and direct finance interventions to improve and expand the financing needs of MSMEs in developing and emerging economies.

### 1.2 The intervention

The policy interventions that have been implemented to alleviate the access constraint include:

(i) legal and regulatory interventions; (ii) financial infrastructure components designed to address information asymmetry and reduce transaction costs; (iii) public support mechanisms; and (iv) private sector models suited to provide sustainable financial services to SMEs (IFC, 2010). The public support schemes and private sector initiatives are made up of a diverse set of programs that fall into numerous categories and are further divided into subcategories. Examples of these various interventions to expand SME finance that have been established to directly target SMEs are establishing or expanding financial infrastructure, partial guarantee schemes, commercial banking models, and other private sector initiatives (IFC, 2013). Furthermore, countries usually employ several interventions that appear to be part of a complete strategy aimed at creating a more conducive environment for SMEs by coordinating and addressing SME development initiatives in a holistic manner. These MSME finance interventions have an impact on the amount of financing and/or investment available; MSME firm performance (e.g., profit, sales, assets, business expansion, indebtedness, survivability, and technology adoption); employment and wages; poverty; financial knowledge or literacy; and women entrepreneurship (Asian Development Bank [ADB], 2021).

### 1.3 Description of the condition

SMEs that have difficulties accessing formal credit, especially long‐term credit, which hinders their ability to develop and innovate, severely limiting their growth potential.

### 1.4 Description of the intervention

A literature review organized on financial interventions that aim to improve MSME's access to finance revealed that the interventions can be classified into categories and outlined below:

- Strategy, legislation, and regulation—interventions that implement national financial inclusion strategies; financial sector legislations and regulations/legal and regulatory framework for payments; and financial consumer protection. This category includes a set of strategies aimed at giving consumers and businesses access to and information to financial products and services. This category also includes legal and regulatory framework that governs banking, insurance, leasing, factoring, and security, as well as secondary regulations and recommendations. Furthermore, it encompasses rules governing liability and recourse, disclosure, and data privacy, and security for financial consumer protection.
- Systems and financial institutions—interventions include reforms in the formal financial system and microfinance institutions; establishment of mobile money agents; use of venture capital funds; engaging in peer‐to‐peer lending; and joining savings clubs/self‐help groups. Institutional reforms to help MSMEs in obtaining financing from both formal and informal sources are included in this category. This category also includes interventions that support MSME financial operations and saving activities.
- Facilitating access—interventions like bank linkages; pitching events and competitions; credit guarantee schemes; digital financial services; informal financial agencies; collateral registries; and credit reporting systems help improve MSMEs access to finance. These interventions create opportunities for MSMEs to connect with formal and informal sources as well as the use of financial technologies to facilitate payments. In addition, these interventions include systems that would use alternate data sources to assess borrower's creditworthiness.
- Lending instruments/finance products—interventions that allow MSMEs to secure microcredit; line of credit; savings; equity; grants; supply chain financing; interest‐free bank accounts; trade credit; microinsurance; and microleasing. This category covers financial products and services that allow MSMEs to start operating their businesses primarily with the use of credit.
- Demand‐side interventions—interventions under this category include financial literacy and education programmes; digital literacy; and opening bank accounts. This category equips MSMEs with training and knowledge needed in starting businesses.

Each intervention category has subcategories that have detailed definitions/descriptions in the Supporting Information: Appendix.

### 1.5 How the intervention might work

Several assumptions were made in generating the theory of change for this evidence and gap map (EGM). The research team assumes that: (1) MSMEs are aware of the interventions and are well‐oriented to use them; (2) MSMEs face constraints in accessing financial products and services to improve their businesses such as challenges in the productive use of credit; (3) bureaucratic obstacles to participate in/benefit from the interventions are minimized; (4) interventions are well‐crafted to the target population's context, financial literacy, and education; and (5) external factors affecting intervention design and implementation may have varying outcomes. As mentioned earlier, financial interventions enhance the access to finance of MSMEs. At various levels, policymakers and the private sector intervene to find ways to increase improved banking services, higher deposit rates, and expanded capital access for MSMEs. Some of these institutions even provide novel approaches and initiatives that can be used to address MSMEs’ non‐financialfinancial needs. Regulatory reforms improved financial infrastructure, an enabling environment, increased financial sector competition, and support measures for financial intermediaries are some of the strategies that can help MSMEs enhance their access to finance and operating capacities (IFC, 2013). Ultimately, the increased financial access of MSMEs lead to sound policy and regulatory environment, enhanced financial inclusion, higher enterprise performance, and positive welfare outcomes.

#### 1.5.1 Scope of the EGM: Theory of change

The scope comprises the following: (1) systems showcasing financial framework and financial institution and/or other sources of financial interventions; (2) platforms facilitating access to finance; (3) actual financial products and services; (4) the population of interest; and (5) the outcomes of interest.

##### Systems and institutions or sources of financial interventions

The map includes interventions from different systems and institutions including various public and private organizations that provide financial services or products such as governments, international donors or organizations, microfinance institutions, banks and insurance companies, and organizations that offer financial education and management programs.

For platforms facilitating access to finance and the lending instruments or financial products and services to be included are already discussed in Section (1.4) earlier. These interventions are utilized by MSMEs with the assumptions that they are well‐oriented to manipulate such platforms and products designed for them and they are facing constraints in terms of access, knowledge, and awareness.

##### Population/MSMEs as beneficiaries

The target population groups included in this map are low‐ and middle‐income countries following the World Bank classification, micro‐, small‐, and medium‐sized enterprises, households and/or single‐person enterprise owner, and farming households and/or farmers. These also include individuals or households who intended to start a business.

**Figure 1:**
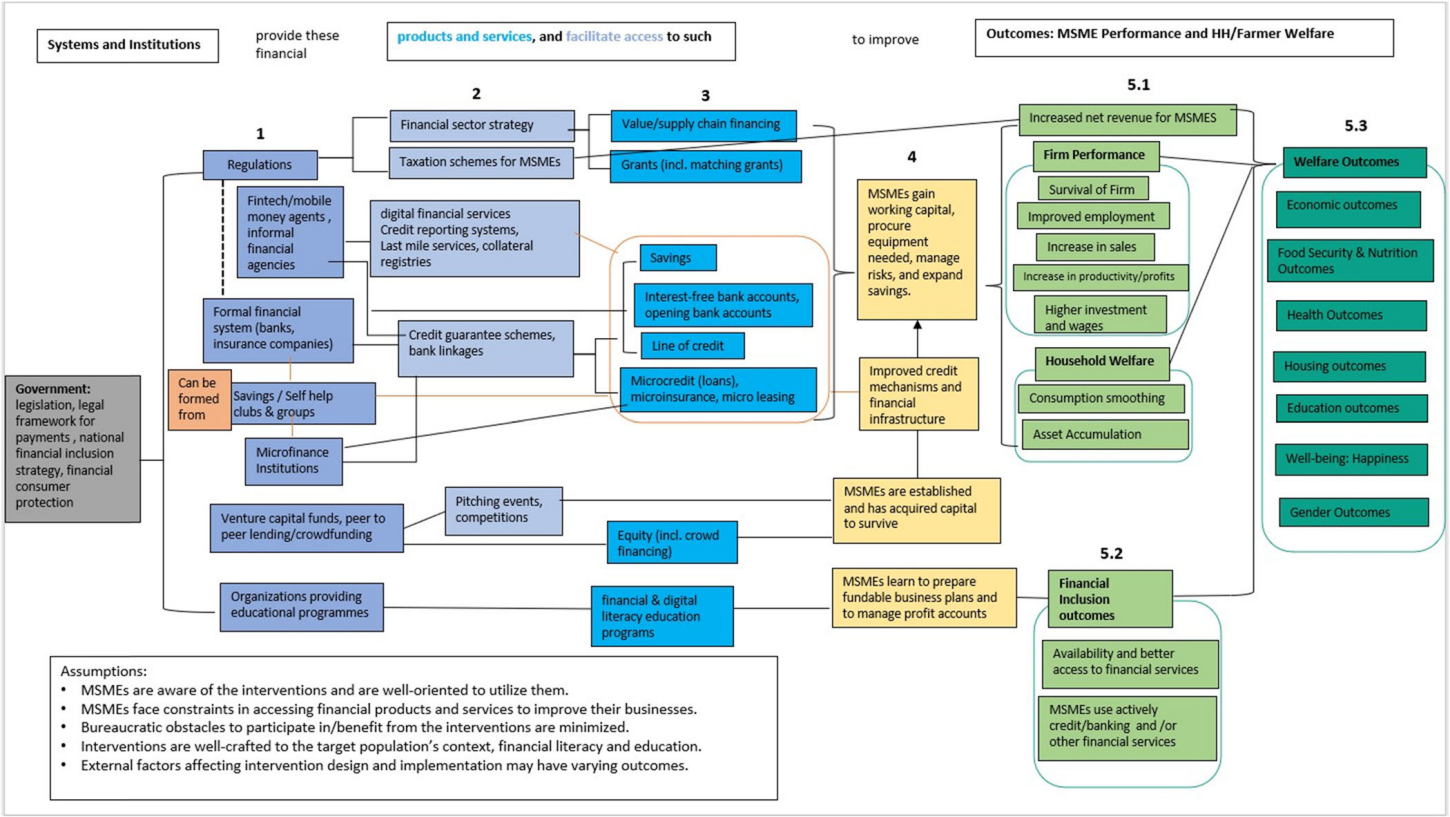
Theory of change for interventions improving access to finance.

The theory of change presents how different levels of the various array of financial interventions from the systems and institutions which are offering platforms that facilitate access and/or directly providing lending instruments or financial products and services improve access to finance. These interventions are shown as the three columns in different shades of blue boxes in Figure 1 (marked as 1 and 2). The theory of change figure shown was developed by the authors as the part of the implementation of this EGM.

- From the government regulations providing financial sector strategy, value or supply chain financing and grants including matching grants help MSMEs gain working capital, procure equipment needed and manage risks.
- From various legal and regulatory framework or simply regulations (represented by the dotted lines) surrounding formal financial institutions like banks and insurance companies, and microfinance institutions and savings or self‐help groups, even financial technologies facilitate access to support MSMEs through different financial products such as savings, interest‐free bank accounts or new bank accounts, line of credit, and microcredit (loans) as well as microinsurance and microleasing. These products can also be provided by or from banks and insurance companies, savings clubs or self‐help groups, and microfinance institutions, as well as digital financial platforms as represented by the orange solid lines. Line of credit, microcredit (loans), and microinsurance are also typically channeled through credit guarantee schemes and bank linkages.
- Some systems like venture capital funds, peer‐to‐peer lending or crowd funding organize pitching events and competitions and offer equity including crowd financing to aid establishment of MSMEs through acquired capital to start a business and/or survival.
- Some organizations providing educational programmes cater to financial literacy training and other educational programs to assist MSMEs in preparing fundable business plans and manage profit accounts.
  As MSMEs are equipped with resources, knowledge, and platforms to improve access to finance, some intermediate outcomes are gained such as working capital, managing risks and challenges, expansion of savings, and improved financial mechanisms (represented by the yellow boxes of Figure 1). These in effect lead to various outcomes on policy and regulatory environment, financial inclusion, enterprise performance, and welfare.
- *Policy and regulatory environment*: include inclusive finance strategies and policy and practice, regulatory framework, and institutional capacity. These outcomes refer to indicators of clear and standardized rules, policies, on how financial institutions should operate, reforms to support enabling environment and mechanisms to develop business models for improving access to finance.
- *Financial inclusion*: such outcomes comprise of financial and digital literacy, availability of financial services, access to financial services, active use of credit or banking services, and use of other financial services. These indicate that MSMEs have better access to financial services and products and are literate and well‐oriented on how these products and services support them. These outcomes in turn lead to different welfare outcomes (Figure 1: marked as 5.2 in light green boxes).
- *Enterprise or Firm performance*: such outcomes include firm survival, improved employment and wages, increase in sales, net revenue for MSMEs, and profits, increase in productivity and higher investments. Some indicators also refer to improvement in management practices such as expenditure on research and development and establishment or creation of firms. These firm performance measures also lead to some welfare outcomes (Figure 1: marked as 5.1 in light green boxes).
- Welfare outcomes: these outcomes cover economic, food security and nutrition, health, education, housing, well‐being, and gender outcomes. Economic outcomes refer to indicators of economic activities such as investments, consumption/expenditure, growth, and poverty reduction measures. Some financial interventions to MSMEs are towards addressing nutrition needs among households and firms. Health outcomes refer to indicators of health treatment, practices, medical expenditures, as well as including mental health measures. Housing outcomes refer to physical improvements in terms of housing. Education as one of the measures of human development include measures on schooling, level of education of firm owners or even their children, attendance, and education expenditures. Well‐being outcomes include financial worries index, happiness scale, and financial security scale. Lastly, gender outcomes refer to measures of women empowerment, women entrepreneurship, and women as decision‐makers in terms of financial management aspects (Figure 1: 5.1 under household welfare and 5.3 dark green boxes).

#### 1.5.2 Why it is important to develop the EGM

MSME development is a key priority for governments and multilateral development banks such as ADB to foster inclusive and sustainable growth. ADB's operational plan for its Strategy 2030 includes supporting SMEs, inclusive business, and inclusive financing to enable inclusive growth to address persisting poverty and reduce inequalities. Based on a study of the current literature, there have been a significant number of impact evaluation studies and systematic reviews on microfinance or microenterprise programs. There have also been a number of SME‐related systematic reviews, however, the majority of them focused on business support interventions and matching grants rather than SME financing. Thus, this EGM will cover the literature on interventions to increase MSME access to finance, which is followed by a review on financial literacy interventions. This will offer policymakers from the public and private sectors with a complete knowledge base, as SME finance programs account for a significant portion of government and private sector's support for inclusive finance.

Although MSME's are shown to contribute substantially to employment creation and inclusive economic growth, they face particular structural hurdles in access to finance and the lack of finance for investment and working capital is often cited as preventing factor for MSME development (see e.g., IFC, 2017; World Bank Group, 2013).

There are a variety of interventions targeted explicitly at MSMEs which aim to solve the obstacles they face in the traditional finance market and/or provide innovative finance solutions to overcome them. These interventions include supporting financial intermediaries, directly providing financial products and services, or otherwise facilitating access to them, and demand‐side interventions such as training in financial literacy.

The purpose of the map is to consolidate and systematically synthesize the rich evidence base on the effects of access to finance interventions for MSMEs. It could therefore serve as a tool in holistically assessing interventions and their outcomes while at the same time identifying further gaps in the evidence base on MSME financing.

#### 1.5.3 Existing EGMs and/or relevant systematic reviews

While there is a plethora of impact evaluation studies and quite a number of systematic reviews on MSME financing, they are typically limited in scope. For instance, some systematic reviews only assess the effects of certain interventions such as microcredit (Vaessen et al., 2014) and self‐help groups (Brody et al., 2015; Gugerty et al., 2019). An enlarged scope of financial interventions may be present in other studies, but the included population is restricted to either microenterprises (Cui et al., 2013) or small and medium enterprises (Kersten et al., 2017) alone. In other examples, both MSMEs may be fully covered but the assessment is hinged on a sole outcome such as employment creation (Grimm & Paffhausen, 2015). There is one EGM on enables, barriers, and impacts of digital financial services by ICTD (2022) published this June. Nevertheless, the EGM will contribute to a broader overview of and systematically synthesize the evidence of the effects of financial access interventions including these digital financial services but targeted for MSMEs, capturing a wide range of outcome variables.

## 2 OBJECTIVES

The goal of this EGM is to describe the existing evidence on the impacts of various interventions to support and improve MSMEs’ access to finance. The interventions can be divided into three categories including systems and institutions, facilitating access, financial products and services, and demand‐side interventions. Consequently, such interventions lead to fundamental outcomes which include firm‐level outcomes and welfare outcomes such as economic, food security and nutrition, health, housing, education, well‐being, and women empowerment.

The map's specific objectives are as follows:

i. Establish a clear definition of the interventions and outcomes related to interventions to improve access to finance interventions of MSMEs.
ii. Map studies of the impact of the interventions on MSMEs’ access to financing projects/programs based on primary studies and systematic reviews of such studies.
iii. Provide a descriptive overview of interventions, contexts, study designs, and geographical distribution of studies.

This project will generate an online and interactive EGM across various interventions improving MSMEs’ access to finance and their corresponding firm performance and/or welfare outcomes.

## 3 METHODOLOGY

### 3.1 Defining EGMs

An EGM illustrates the complete existing research literature and evidence on a given subject. It is a systematic presentation of the availability of relevant evidence for a particular policy domain (Saran & White, 2018). Similar to systematic reviews, an EGM subscribes to a pre‐specified and published protocol, but it only provides a summary of the evidence, not assess the findings from the studies. Hence, EGM covers a wider scope than systematic reviews.

The final output of an EGM is a research article or report but conveniently, it can also be disseminated through an interactive map plotted as matrix of included studies and their corresponding interventions and outcomes. At first sight, one can check the evidence available or lack thereof for one's subject matter. This EGM on improving access to finance for MSMEs includes evidence from impact evaluations and systematic reviews.

### 3.2 EGM framework

#### 3.2.1 Population

The population in eligible studies are (i) micro, small, and medium‐sized enterprises^1^ including studies focusing on households, small holder farmers and single person enterprise or business owners; and (ii) financial institutions and agencies, and the staff of those institutions and agencies.

For specific filters of the population sub‐groups, the following groups of interest will be coded:

(1) Population sub‐groups include: Women; Youth/young people; Older people; Poor and disadvantaged; Humanitarian settings; people with disabilities; and rural/remote
(2) Type of firm receiving financial intervention include: Micro enterprise including households, farmers, and single person enterprise; Community groups; Small enterprise; and Medium enterprise
(3) MSME sectors included: Agriculture; Construction; Manufacturing; Services; Transportation and communication; Wholesale and retail trade; Others.
(4) Country and Region of study include: low‐ and middle‐income countries or region include lower‐middle‐income countries and upper‐middle‐income countries. High‐ income countries are excluded. These are based on World Bank's income classification of countries and geographical criteria.
(5) Study design which will be coded as experimental, non‐experimental, and systematic review.

##### Intervention

Eligible studies must be studies of an intervention. Studies of financial inclusion in which there is no intervention will not be included.

Interventions are broadly defined to include various approaches to supporting financial inclusion such as legislation and regulatory aspects, systems and institutions that enable financing, facilitating access to finance, different lending instruments or financial products including traditional forms of microcredit, and demand‐side interventions such as programs on financial literacy. We include interventions targeted to micro, small, and medium‐sized enterprises, Micro enterprises includes households, small holder farmers, and single person enterprises.

Table 1 lists the intervention categories and corresponding sub‐categories. Annex X provides a more detailed description of each.

**Table 1:** Intervention categories and sub‐categories.

Intervention categories	Sub‐categories
Strategy, legislation, and regulation	National Financial Inclusion Strategy
	Financial sector legislation and regulations (including tax regime)/Legal and regulatory framework for payments (including insolvency mechanisms)
	Financial consumer protection
Systems and institutions	Formal financial system (banks and insurance companies)
	Microfinance institutions
	Mobile money agents (including remittances)
	Venture capital funds
	Peer‐to‐peer lending/crowdfunding
	Savings clubs and groups/self‐help groups
Facilitating access	Bank linkages
	Pitching events, competitions
	Credit guarantees incl. partial credit guarantee schemes
	Fintech/Digital financial services (mobile and e‐money and banking and payment systems and infrastructure)
	Informal financial agencies or agents incl. last mile interventions;
	Collateral registries
	Credit reporting systems
Lending instruments/financial products	Microcredit (loans)
	Line of credit
	Savings
	Equity (incl. crowd financing)
	Grants (incl. matching grants)
	Supply chain financing
	Interest‐free bank accounts
	Trade credit
	Microinsurance
	Microleasing/hire purchase
Demand‐side interventions	Financial literacy and education programmes
	Digital literacy
	Opening bank accounts for entrepreneurs

##### Outcomes

The map will include outcome domains surrounding policy environment, financial inclusion, firm performance, and welfare. Below are the four outcome domains with their corresponding sub‐categories (Table 2).

**Table 2:** Outcome domains and sub‐categories.

Outcomes	Sub‐categories
Policy and Regulatory Environment	Inclusive finance strategies and policy and practice
	Regulatory framework
	Institutional capacity
Financial Inclusion	Financial and digital literacy
	Availability of financial services
	Access to financial services
	Active use of credit/banking services (including loan volume)
	Use of other financial services
Enterprise/firm performance	Management practices (including Research & Development expenditure)
	Employment and wages
	Sales/revenue, exports
	Productivity/Profits/Return on investments
	Established/Survival
Welfare outcomes	Economic (including employment)
	Food security and nutrition
	Health
	Housing
	Education
	Well‐Being: Happiness
	Gender

### 3.3 Criteria for including and excluding studies

#### 3.3.1 Study design

The EGM includes impact evaluations or systematic reviews covering relevant interventions for a selected target group that fall within the above‐discussed categories. Moreover, eligible studies need to include a methodological design that addresses the potential endogeneity problem due to selection biases concerning the intervention.

Included studies either have quasi‐experimental study designs or other suitable methodological approaches with a potential selection bias awareness. Methodological designs and analytical frameworks that meet this criterion are, for example, natural experiments, randomized controlled trials, regression discontinuity analyses, propensity score matching, difference‐in‐difference approaches, and the use of instrumental variables. Starting from the regression discontinuity design down to instrumental variable method are some of the examples of non‐experimental design methods to be considered.

Before versus after study designs with no suitable comparison group or designs including a naïve selection of a control group without accounting for selection biases are excluded from the EGM. Qualitative evidence or study designs including literature reviews, key informant interviews, focus group discussions, and descriptive analyses are also not considered for the map.

#### 3.3.2 Country specification

Studies included in the EGM are restricted to evaluations on interventions that have been conducted in countries classified by the World Bank as low‐ or middle‐income countries. In systematic reviews, the map may also include studies from high‐income countries if low‐ and middle‐income countries are included.

#### 3.3.3 Status and formalities

For feasibility reasons, the EGM is limited to studies in the English language. There are no restrictions regarding publication status or date of studies, that is, it includes journal articles and online accessible reports or working papers that are not yet published and are ongoing.

### 3.4 Search strategy and status of studies

Since the majority of studies were written in English, over 40 databases and institutional websites will be searched using the standard search approach described in Supporting Information: Appendix B. The reviewers provided a list of institutional websites and journals that focus on financial inclusion as well as the coding sheet to an information science specialist. The said specialist in turn provided, pre‐tested, and reviewed the search strategy. The search terms reflect the keywords used in prior reviews and further refined using terminology that was reflective of MSMEs, access to credit interventions, study designs, and developing countries. Depending on the database, search terms will be typically consisted of three blocks of terms and, if permitted, the necessary Boolean or proximity operators. Terms relating to (1) intervention, (2) study design, (3) LMICs, and (4) MSMEs as detailed in Supporting Information: Appendix B, will be included in blocks. While institutional websites mostly featured gray literature in the form of evaluation reports and working papers, scholarly databases contained peer‐reviewed articles. All identified studies will undergo title, abstract, and full text screening. The studies that will be found in the search will be put into EPPI Reviewer 4 for coding and screening.

Below are the search strategies that will be used to filter out potential studies for the map.

Database searches will include: Agris, Repec, Academic Search, ScienceDirect, EconPapers, Econstor, Taylor & Francis Online, Jstor, Scielo, Ebsco Discovery, SpringerLink, and Emerald Insight.

Organization searches will include 3ie impact evaluation repository, Economic Research Institute for ASEAN and East Asia, World Bank e‐library, Banco Bilbao Vizcaya Argentaria (BBVA) Research, International Financial Corporation (IFC), International Monetary Fund (IMF), Social Protection Organization, Massachusetts Institute of Technology's digital repository, Innovation Growth Lab, Global Partnership for Financial Inclusion, Institute of Labor Economics (IZA), Centre for Economic Policy Research, Innovations for Poverty Action, Alliance for Financial Inclusion (AFI), SME Finance Forum, Organization for Economic Cooperation and Development (OECD) library, Asian Development Bank (ADB), Asian Development Bank (ADBI), Inter‐American Development Bank (IADB), Poverty Action Lab, Center for Effective Global Action (CEGA), United Nations University World Institute for Development Economics Research (UNU‐WIDER), National Bureau of Economic Research, and VoxDev.

In additional to the database and institutional website search, the advisory group recommended to manually search journals dated in the last 5 years. Journal searches will include: *International Business & Economics Research Journal (IBER)*, *Journal of Economics and Behavioral Studies*, *International Small Business*, *Journal of Small Business Economics*, *The Journal of Finance*, *Journal of Banking and Finance*, *Journal for SME Development*, *Review of Development Finance*, *American Economic Review*, *Journal of Entrepreneurship and Small Business*, *Journal of Asian Finance, Economics and Business*, *SSRN Electronic Journal*, *World Development*, *Journal of Development Economics*, *Journal of Development Studies*, *Journal of Development Effectiveness*, *Journal of Innovation and Entrepreneurship*, *American Economic Review*, and *Journal of Business Venturing*.

Bibliographic searches: We will screen the systematic reviews to locate additional primary studies. To identify additional primary studies and systematic reviews, we will also conduct bibliographic back‐referencing of reference lists of all included systematic reviews.

Supporting Information: Appendix B presents the search strings used for publication databases and search engines, with terms for interventions, regions, and approaches.

### 3.5 Screening and selection of studies

At both the title/abstract and full‐text screening stages, we will use EPPI reviewer to assess studies for inclusion. Each full‐text and title/abstract will be screened by two researchers. Discussion will be used to address any differences about inclusion.

### 3.6 Data extraction, coding, and management

For included studies, a data extraction tool will be used to capture descriptive data from impact evaluations and systematic reviews. The descriptive data will include all the necessary information required to generate the map such as intervention type, outcome, study design, firm types, and firm sectors among others.

Data extraction tool or coding framework is presented in Supporting Information: Appendix A. A total of four reviewers will conduct the data extraction independently, two reviewers per study, and two from each will be the third‐party arbiter to resolve the disagreements. The four reviewers are trained with the tool through piloting sessions before the commencement of the coding stage. This ensures consistency in coding and resolves any issues or ambiguities.

#### 3.6.1 Unit of analyses

If some studies are published in one or more types of publications such as working paper and a journal article, the most recent paper will be included in the map. Some systematic reviews are also published in two or more ways, but the Campbell review version is considered in the map.

#### 3.6.2 Planned analyses and presentation

The coding framework and coding tool which includes the filters are presented in Supporting Information: Appendices A and C. The map will showcase the default matrix of intervention categories against the outcome categories. Other versions of the map will include types of firm receiving the intervention against the intervention categories and outcome categories as well; population filters against intervention and outcome categories; and country by region against intervention categories. The report will describe the evidence according to these intervention categories, outcome categories, and the firm types. Summary tables on the characteristics of the included studies will also be included. A narrative summary of the results of the systematic reviews will also be presented.

Filters in the EGM include the scope or geographical coverage, study design, intervention, and outcome sub‐categories, country by income, country by region, firm sectors, population, and publication status.

### 3.7 Quality appraisal

We will not use a critical appraisal tool for the primary studies and systematic reviews but coding on study design or methodology is covered in the data extraction tool. We will be doing a narrative synthesis of the systematic reviews included in the map as alternative.

## 4 STAKEHOLDER ENGAGEMENT

The existing proposed framework will be reviewed by an Advisory Group comprising of the following:

1. Representatives from the Alliance for Financial Inclusion
2. Dr. Maren Duvendack (author of reviews on financial inclusion and microcredit)
3. Mike Joyce (SME financial inclusion practitioner)
4. Dr. Masato Abe (UNESCAP)
5. ADB finance sector staff

We have gathered colleagues from financial sector organizations and other experts/authors on the topic of MSME finance. As we finished generating the EGM, we will reach out to the same panel of experts for their feedback and comments.

## ROLES AND RESPONSIBILITIES

- *Content expertise*:
  Prof. Robert Lensink is a professor of finance and financial markets at the University of Groningen. Alyssa Villanueva is a consultant at Asian Development Bank's Sustainable Development and Climate Change Finance Sector Group. She works as a research analyst for projects related to SME credit guarantee schemes, SME lending schemes, and banking sector non‐performing loans.
- *Systematic review method expertise*:
  Most of the authors are experienced systematic reviewers, which means that they are proficient in conducting various processes in an EGM, such as screening, quality assessment and coding.
  Howard White will provide technical support for the conducting the review.
- *EGM methods expertise*:
  Howard White as CEO provides technical and strategic support for the development of EGM in Campbell library. Most of the team members have previous experience in systematic review methodology, including search, data collection, statistical analysis, theory‐based synthesis, which mean they are proficient in carrying out the various processes in an EGM, such as search, eligibility screening, quality assessment and coding.
- *Information retrieval expertise*:
  The authors will be supported by information retrieval specialist, Dr. John Eyers, on an as‐ needed basis. John Eyers is a trained information retrieval specialist and has experience of supporting over 50 systematic maps and reviews in social sciences areas.

## SOURCES OF SUPPORT

This EGM is supported by the Asian Development Bank's Independent Evaluation Department.

## DECLARATIONS OF INTEREST

No conflicts of interest.

## PRELIMINARY TIMEFRAME

The draft map will be generated in March 2022, and the final version of the map by April or May 2022.

## PLANS FOR UPDATING THE EGM

We plan to update the map (or support others in doing so) when sufficient further studies and resources become available.

## Supporting information

Supporting information.

## Footnotes

1 This EGM follows the definition of the International Financial Corporation (IFC) wherein it defines a business as a micro, small, or medium enterprise (MSME) if it satisfies two of the three criteria listed there (employees, assets, and sales), or if the loan it has received fits within the appropriate MSME loan size proxy. See more at https://www.ifc.org/wps/wcm/connect/industry_ext_content/ifc_external_corporate_site/financial+institutions/priorities/ifcs+definitions+of+targeted+sectors.
