# Supplementary material for: PROTOCOL: Effects of interventions to improve access to financial services for micro‐, small‐ and medium‐sized enterprises in low‐ and middle‐income countries: An evidence and gap map
Source: Campbell Syst Rev. 2023 Jul 5;19(3):e1341. doi: 10.1002/cl2.1341 (PMC10320698; doi:10.1002/cl2.1341)
Supplement: Supplementary file 1 — Supporting information. [file CL2-19-e1341-s001.docx]

**Appendix A: Coding framework**

**Intervention categories and sub-categories**

| **Interventions** | | **Sub-categories** | | **Definitions/Descriptions** | |
| --- | --- | --- | --- | --- | --- |
| Strategy, legislation and regulation | | National Financial Inclusion Strategy | | A set of strategies to provide individuals and businesses with access to and informed use of a wide range of high-quality and affordable savings, credit, payment, insurance, and investment products and services that meet their needs: (1) widespread and accessible delivery channels; (2) diverse, innovative, and customer-centric products for individuals; (3) accessible and supportive financial services; and (4)  financial consumer protection. | |
|  |  | Financial sector legislation and regulations (including tax regime) / Legal and regulatory framework for payments (inc.  insolvency mechanisms) | | The legal and regulatory framework in a specific country creates the rules by which all financial institutions, instruments, and markets operate. It covers laws governing banking, insurance, leasing, factoring, and security, as well as secondary rules and guidelines. | |
|  |  | Financial consumer protection | | rules governing liability and recourse, disclosure, and data privacy and security | |
| Systems and institutions | | Formal financial system (banks and insurance companies) | | Interventions serving MSMEs to increase competition and market diversification that can: (i) reduce information asymmetry of SMEs and improve risk management; (ii) reduce the cost of lending by improving the efficiency of bank operations; (iii) develop products better adapted to the needs of SMEs; (iv) improve financial services for SMEs; and  (v) cooperate with SME organizations and other business development providers | |
|  |  | Microfinance institutions | | Interventions serving MSMEs to: (i) ensure finances for low-income borrowers; (ii) empower women by financing micro, small, and medium-sized businesses; and (iii) provide rural women with access to education and finance; (iv) reconstruct post- conflict communities and resuscitate women’s livelihoods; (v) help businesses and livelihoods  outside of capital cities; and (vi) nurture small enterprises to help diversify economies. | |
|  |  | Mobile money  agents (including remittances) | | supports critical financial operations such as money  transfers and bill payments by converting e-money to cash. | |
|  |  | Venture capital funds | | Investments made during the launch stages of a business in exchange for a share in the company, usually consists of at least 10 to 20 partners. | |
|  |  | Peer to peer lending  / crowdfunding | | Lending money to individuals or businesses via online services that connect lenders and borrowers, attempting to operate at a lower cost and deliver services at a lower cost than traditional financial institutions. | |
|  | | Savings clubs and groups/SHGs | | A typical SHG has 12 to 30 members (Rutherford, 2000). They participate in collective bargaining, risk spreading, and peer education and social assistance, among other activities. A typical savings group, on the other hand, consists of 10-20 members, the majority of whom are women. Aside from offering reliable savings mechanisms and improving credit access, savings organizations also promote income-  generating activities, provide alternate kinds of insurance, and increase participants’ social capital. | |
| Facilitating access | | Bank linkages | | ties with their MFIs and lending institutions that  could assist them in expanding their understanding of accessible financial services | |
|  |  | Pitching events, competitions | | Training that help entrepreneurs evaluate their businesses, the commercialization concept, and expected revenue streams, as well as undertake a market sizing exercise and characterize their competitive positioning. Mentoring and pitch  training are also given to help owners prepare for pitch competitions and events. | |
|  |  | Credit guarantees inc. Partial Credit Guarantee Schemes | | Credit guarantee schemes are arrangements in which a third party, known as the guarantor, agrees to repay the lender part or all of the loan amount if the borrower defaults. The guarantor assumes some or all of the credit risk, decreasing the risk faced by financial intermediaries and allowing businesses to  acquire credit or enhance their borrowing terms and conditions. | |
|  |  | Fintech/ Digital financial services (mobile and e- money and banking and payment  systems and infrastructure) | | permits mobile banking, the settlement of (international) payments, and the collection and use of alternate data sources for creditworthiness assessments of SMEs, among other things. | |
|  |  | Informal financial agencies or agents including last mile interventions | | Funds sourced outside the regulatory framework and enforcement of a central banking and finance authority such as personal savings, borrowing from relatives, or ‘loan sharks' | |
|  |  | Collateral registries | | A registry where individuals and businesses can register their movable assets (such as inventory, accounts receivables, crops, and equipment) as collateral for loans in order to obtain credit from lenders. Lending will be less risky because lenders will have collateral to fall back on if a borrower  defaults on payments, and interest rates on loans are expected to fall. | |
|  |  | Credit reporting systems | | Makes historical credit data and other important information on large firms and individuals widely available to address information asymmetries. | |
| Lending instruments/finance products | | Microcredit (loans) | | are also known as microloans or the small working capital loans provided by microfinance institutions to low-income individuals or organizations who are traditionally excluded from regular banking. | |
|  |  | Line of credit | | a contract between a bank and a borrower that establishes a maximum loan balance that the bank will allow the borrower to keep. The client only pays interest on the portion of the credit line that is used.  After the money is repaid, the funds can usually be borrowed again. | |
|  | | Savings | | Deposit and savings products used as basic financial management tools to help them organize their earnings and savings. | |
|  |  | Equity (including crowd financing) | | Securities in a private enterprise (company) that is not listed on stock exchanges are sold to raise funds from the crowd (via an internet platform). It’s similar to debt crowdfunding, however instead of raising loan cash, the company raises equity capital by  selling stocks. | |
|  |  | Grants (including matching grants) | | Costs of training, marketing, and/or visiting trade fairs covered by the government | |
|  |  | Supply chain financing | | The use of financing and risk mitigation products/tools such as contracts, receivables discounting, factoring, payables finance (reverse factoring), distributor finance, and purchase-order finance to optimize the management of working capital and liquidity invested in supply chain  processes and transactions | |
|  |  | Interest free bank accounts | | bank account with a zero percent interest rate | |
|  |  | Trade credit | | Buyers can buy products or services on credit and pay the supplier later, i.e. a supplier provides a short- term loan to a buyer. | |
|  |  | Microinsurance | | Insurance provided by MFIs, cooperatives, and NGOs to small and micro entreprenuers as a risk  management tool and social protection product | |
|  |  | Microleasing/hire purchase | | External asset-based financing where SMEs lease an asset to generate cash flows or substitute to collateral | |
| Demand-side interventions | | Financial literacy and education programmes | | Basic accounting and management information and tools are supplied to MSMEs, allowing them to grow and acquire the necessary business skills and procedures for prudent and effective management. By educating and organizing a team of skilled SME Relationship Managers, the programs could take the  shape of seminars, workshops, and/or on-the-job practical training. | |
|  |  | Digital literacy | | Training on how to conduct and exploit business prospects using e-business technology in order to achieve higher entrepreneurial success. | |
|  |  | Opening bank accounts for  entrepreneurs | | Eliminating discriminatory banking laws and simple account opening process/requirements | |

**Outcome categories and sub-categories**

| **Outcomes** | **Sub categories** | **Definitions/Descriptions** |
| --- | --- | --- |
| Policy and Regulatory Environment | Inclusive finance strategies and policy and practice | refer to rules, policies, and conventions on how banks should operate; strategies with a vision of serving all members of society at its best, including the underserved, to have |
|  |  | access to and usage of quality and essential financial services (Rahman, 2017) |
|  | Regulatory framework | provides a clear, standardized understanding of MSMEs within the national context and facilitates technical assistance and channel financial benefits and other policy incentives more effectively (AFI 2016; AFI 2021); This also includes regulatory reforms to support enabling environment, strengthen financial infrastructure, and implement public and  private initiatives tailored for MSMEs (IFC 2013); |
|  | Institutional capacity | refers to institutional mechanisms and tools to develop business model for targeting access to finance, prepare or support for business recovery, strengthen business support functions including staff capacity, and  improve coordination and awareness of available MSME support (ADB 2020) |
| Financial Inclusion | Financial and digital literacy | “the combination of awareness, knowledge, skills, attitudes and behaviour that a potential entrepreneur or an owner or manager of a micro, small or medium sized enterprise should have in order to make effective financial decisions to start a business, run a  business, and ultimately ensure its sustainability and growth” (OECD 2018) |
|  | Availability of financial services | enable MSMEs to have access to financial services through indicators such as the existence of savings, credit, and payment services in banks and in regulated nonbank financial institutions i.e. loan to banks to expand lending services to SMEs (IFC 2019); if the business owner has a bank account or  not; no. of banks; no. of households who open bank accounts |
|  | Access to financial services | refers to indicators posing that MSMEs either have access or do not have access to financial services such as numbers of people, households, and firms saving, receiving credit, making payments, and using other financial products from various sources, both formal and informal (ideally). (World Bank 2009) such as the value of bank deposits, mortgage  payments, insurance for payments |
|  | Active use of credit/banking services (including loan volume) | refers to the use of different types of credit (ie. loan volume and value) and banking services such as savings accounts, checking accounts,  debit and credit cards, insurance and wealth management over a period of time |
|  | Use of other financial services | refers to use of other financial services from other providers aside from banking services such as digital financial services which include  mobile money operators, e-money issuers, branchless services (AFI 2019) |
| Enterprise performance | Management practices- R&D expenditure | refers to management practices attributed to financial interventions or increasing access to finance such as research and development expenditure to promote investments, participation to SME programs that provide access to financial services, and financing  programs for debt restructuring and financial incentives. (Lopez-Acevedo & Tan 2011) |
|  | Employment and wages | refers to employment indicators that represent enterprise or firm growth including employment size, employment rates, no. of wage and salary workers, and casual workers, wages, increase in skilled-self-employment, employment/self-employment hours  (McKenzie 2015; Lopez-Acevedo & Tan 2011; Banerjee et al 2020) |
|  | Sales/revenue, profits, exports | include measures of lagged sales, sales growth, revenue (or output) and, exports as percentage of sales, can also be expressed as annual sales of goods and services; Profit in simplest terms is the difference of the costs of  goods sold from the sales revenue. |
|  | Productivity/Returns on Investment | includes labor productivity calculated as total revenue per number of employees in real terms (Colacelli & Hong 2019); and total factor productivity measures how efficiently the production factors of capital and labor are combined in generating value added (Erken et al 2016);. returns on investment refer to the  amount of return on a particular investment relative to its costs. |
|  | Established/Survival | means an enterprise or firm has been established or created (Banerjee et al 2020); and necessity driven enterpreneurship  meaning as a means of survival (Omidyar Networ 2013; McKenzie 2015) |
|  | Input adoption practices | refer to input usage, input purchases or buying of inputs for investments or reinvestments of the firms or businesses |
| Welfare outcomes | Economic (including employment) | indicators of various economic activities including assets (i.e. asset value, asset index, average change in fixed assets), investments and consumption/expenses (monetary amount), output, growth, poverty reduction measures such as income (i.e. per capita income level change, % poverty status), job creation or employment; also includes financial capital-ability to buy time, undertake more ambitious strategies, change courses of actions, and meet the financing demands imposed by growth”, thus aiding both the  survival and the growth of new ventures (Cooper et al., 1994: 391) |
|  | Food security and nutrition | indicators that reflect that funds from financial interventions to MSMEs were allocated to pressing nutritious needs  including selection of nutritious foods (i.e. energy intake-kcal/day (Chliova et al 2015) |
|  | Health | indicators that reflect improvement in different health measures brought about by funds earned from MSME’s operations such as health knowledge, treatment, practices, medical expenditures, mental health (i.e. % reporting illness, % knowledge of pre-natal care or medical check-up; % with at least 1 immunization per child, % of women who sought formal care in the event of illness;  health expenditure per capita, % reporting emotional stress) |
|  | Housing | one of the welfare indicators aside from income and expenditure wherein measures include whether households have made physical improvements- roofing etc, with their houses over a certain period of time (Berhane et al 2011); housing quality which refers to the materials like tile roof and concrete houses indicating highest quality, and other housing  characteristics (Brannen 2010) |
|  | Education | one of indicators of human development which includes various education measures such as improved education of firm owners’ children, access to schooling, willingness and ability to send children to school, and adults’ education (i.e. years of schooling of family  workers, school attendance, education expenditure) |
|  | Well-Being: Happiness | measures of well-being, indicated by worries and happiness reflected from financial interventions to MSMEs (i.e. worries index,  financial worries index, happiness scale, financial security scale) |
|  | Gender | one of the indicators of human development which includes women empowerment (i.e. measured as independence- % allowed to make decisions regarding purchase of assets; discrimination (Brock & De Haas 2020)), “the expansion in people’s ability to make strategic life choices in a context where this ability was previously denied to them; a process that entails thinking outside the system and challenging the status quo, where people can make choices from the vantage point of real  alternatives without punishingly high costs,” (Kabeer 1999; Brody et al 2015); |

**Appendix B: Search terms Intervention search term**

TI ( ( micro-financ* OR “micro financ*“ OR microfinanc* OR micro-loan* OR microloan* OR “micro loan*“ OR microleas* OR micro-leas* OR “micro leas*“ OR microlending OR micro-lending OR “micro lending” OR microinsurance OR micro-insurance OR “micro insurance” OR “microgroup lending” OR microfranchis* OR micro-franchis* OR “micro franchis*“ OR “micro credit*“ OR microcredit* OR micro-credit* OR saving* OR grant OR grants OR equity OR micro-saving* OR microsaving* OR “Smallholder financ*“ OR “rural financ*“ OR “rural credit” OR ROSCAs OR SHGs OR “group lending” OR “community saving*“ or “small loan*“ or “small lend*“ or ((bank or credit*) N3 cooperat*) or ((credit or loan* or lend*) N3 program*) or (community N3 (bank* or saving* or loan* or lend*)) or “income generat*“ or grameen OR ROSCA* OR stokvel* OR ((financ* OR economic) N2 (literacy OR education OR skills OR training OR knowledge OR capab*)) OR banking OR budgeting OR “financial inclus*“ OR crowdfund* or “crowd fund*“ or (peer-to-peer N2 (lend or loan*)) or (“venture capital” N2 fund*) OR (financ* N4 (legislat* or law or laws or legal* or “supply chain” or “last mile” or crowd or informal or regulat* or (consumer* N3 protect*) or tax*)) OR (insolvency N2 mechanism*) OR “money manag*“ OR “credit guarantee*“ OR (bank* N3 linkage*) OR e-money OR “informal financ* agen*“ OR “collateral registr*“ OR (“credit report*“ N3 system*) OR “trade credit” OR (interest-free N3 bank*) OR rationing OR earmarking OR “bank account*“ OR “lock box*“ OR ((saving* OR self-help) N3 (club OR clubs OR group*)) OR “saving box*“ OR ((access* OR participat*) N3 ( financ* OR credit OR saving* OR loan* OR lending)) OR fintech OR “mobile monies” OR M-PESA OR “mobile banking” OR cashless)) OR AB ( (micro-financ* OR “micro financ*“ OR microfinanc* OR micro-loan* OR microloan* OR “micro loan*“ OR microleas* OR micro-leas* OR “micro leas*“ OR microlending OR micro-lending OR “micro lending” OR microinsurance OR micro- insurance OR “micro insurance” OR “microgroup lending” OR microfranchis* OR micro- franchis* OR “micro franchis*“ OR “micro credit*“ OR microcredit* OR micro-credit* OR saving* OR grant OR grants OR equity OR micro-saving* OR microsaving* OR “Smallholder financ*“ OR “rural financ*“ OR “rural credit” OR ROSCAs OR SHGs OR “group lending” OR “community saving*“ or “small loan*“ or “small lend*“ or ((bank or credit*) N3 cooperat*) or ((credit or loan* or lend*) N3 program*) or (community N3 (bank* or saving* or loan* or lend*)) or “income generat*“ or grameen OR ROSCA* OR stokvel* OR ((financ* OR economic) N2 (literacy OR education OR skills OR training OR knowledge OR capab*)) OR banking OR budgeting OR “financial inclus*“ OR crowdfund* or “crowd fund*“ or (peer-to-peer N2 (lend or loan*)) or (“venture capital” N2 fund*) OR (financ* N4 (legislat* or law or laws or legal* or “supply chain” or “last mile” or crowd or informal or regulat* or (consumer* N3 protect*) or tax*)) OR (insolvency N2 mechanism*) OR “money manag*“ OR “credit guarantee*“ OR (bank* N3 linkage*) OR e- money OR “informal financ* agen*“ OR “collateral registr*“ OR (“credit report*“ N3 system*) OR “trade credit” OR (interest-free N3 bank*) OR rationing OR earmarking OR “bank account*“ OR “lock box*“ OR ((saving* OR self-help) N3 (club OR clubs OR group*)) OR “saving box*“ OR ((access* OR participat*) N3 ( financ* OR credit OR saving* OR loan* OR lending)) OR fintech OR “mobile monies” OR M-PESA OR “mobile banking” OR cashless)) OR SU ( (micro-financ* OR “micro financ*“ OR microfinanc* OR micro-loan* OR microloan* OR “micro loan*“ OR microleas* OR micro-leas* OR “micro leas*“ OR microlending OR micro-lending OR “micro lending” OR microinsurance OR micro-insurance OR “micro insurance” OR “microgroup lending” OR microfranchis* OR micro-franchis* OR “micro franchis*“ OR “micro credit*“ OR microcredit* OR micro-credit* OR saving* OR grant OR grants OR equity OR micro- saving* OR microsaving* OR “Smallholder financ*“ OR “rural financ*“ OR “rural credit” OR ROSCAs OR SHGs OR “group lending” OR “community saving*“ or “small loan*“ or “small lend*“ or ((bank or credit*) N3 cooperat*) or ((credit or loan* or lend*) N3 program*) or (community N3 (bank* or saving* or loan* or lend*)) or “income generat*“ or grameen OR ROSCA* OR stokvel* OR ((financ* OR economic) N2 (literacy OR education OR skills OR training OR knowledge OR capab*)) OR banking OR budgeting OR “financial inclus*“ OR crowdfund* or “crowd fund*“ or (peer-to-peer N2 (lend or loan*)) or (“venture capital” N2 fund*) OR (financ* N4 (legislat* or law or laws or legal* or “supply chain” or “last mile” or crowd or informal or regulat* or (consumer* N3 protect*) or tax*)) OR (insolvency N2 mechanism*) OR “money manag*“ OR “credit guarantee*“ OR (bank* N3 linkage*) OR e-money OR “informal financ* agen*“ OR “collateral registr*“ OR (“credit report*“ N3 system*) OR “trade credit” OR (interest-free N3 bank*) OR rationing OR earmarking OR “bank account*“ OR “lock box*“ OR ((saving* OR self-help) N3 (club OR clubs OR group*)) OR “saving box*“ OR ((access* OR participat*) N3 ( financ* OR credit OR saving* OR loan* OR lending)) OR fintech OR “mobile monies” OR M-PESA OR “mobile banking” OR cashless))

TI ( ( SHGs OR “group lending” OR “community saving*“ or “small loan*“ or “small lend*“ or ((bank or credit*) N3 cooperat*) or ((credit or loan* or lend*) N3 program*) or (community N3 (bank* or saving* or loan* or lend*)) or “income generat*“ or grameen OR ROSCA* OR stokvel* OR ((financial OR economic) N2 (literacy OR education OR skills OR training OR knowledge OR capab*)) OR banking OR budgeting OR “money manag*“ OR “consumption smoothing” OR rationing OR earmarking OR “bank account*“ OR “youth account*“ OR “lock box*“ OR “piggy bank*“ OR “saving box*“ OR “financial inclusi*“ OR “inclusive financ*“ OR fintech OR “mobile monies” OR M-PESA OR “mobile banking” OR cashless)) OR AB ( (SHGs OR “group lending” OR “community saving*“ or “small loan*“ or “small lend*“ or ((bank or credit*) N3 cooperat*) or ((credit or loan* or lend*) N3 program*) or (community N3 (bank* or saving* or loan* or lend*)) or “income generat*“ or grameen OR ROSCA* OR stokvel* OR ((financial OR economic) N2 (literacy OR education OR skills OR training OR knowledge OR capab*)) OR banking OR budgeting OR “money manag*“ OR “consumption smoothing” OR rationing OR earmarking OR “bank account*“ OR “youth account*“ OR “lock box*“ OR “piggy bank*“ OR “saving box*“ OR “financial inclusi*“ OR “inclusive financ*“ OR fintech OR “mobile monies” OR M-PESA OR “mobile banking” OR cashless)) OR SU ( (SHGs OR “group lending” OR “community saving*“ or “small loan*“ or “small lend*“ or ((bank or credit*) N3 cooperat*) or ((credit or loan* or lend*) N3 program*) or (community N3 (bank* or saving* or loan* or lend*)) or “income generat*“ or grameen OR ROSCA* OR stokvel* OR ((financial OR economic) N2 (literacy OR education OR skills OR training OR knowledge OR capab*)) OR banking OR budgeting OR “money manag*“ OR “consumption smoothing” OR rationing OR earmarking OR “bank account*“ OR “youth account*“ OR “lock box*“ OR “piggy bank*“ OR “saving box*“ OR “financial inclusi*“ OR “inclusive financ*“ OR fintech OR “mobile monies” OR M-PESA OR “mobile banking” OR cashless))

TI ( (micro-financ* OR “micro financ*“ OR microfinanc* OR micro-loan* OR microloan* OR “micro loan*“ OR microleas* OR micro-leas* OR “micro leas*“ OR microlending OR micro-lending OR “micro lending” OR microinsurance OR micro-insurance OR “micro insurance” OR “microgroup lending” OR microfranchis* OR micro-franchis* OR “micro franchis*“ OR “micro credit*“ OR microcredit* OR micro-credit* OR saving* OR micro- saving* OR microsaving* OR “Smallholder financ*“ OR “rural financ*“ OR “rural credit”)) OR AB ( (micro-financ* OR “micro financ*“ OR microfinanc* OR micro-loan* OR microloan* OR “micro loan*“ OR microleas* OR micro-leas* OR “micro leas*“ OR microlending OR micro-lending OR “micro lending” OR microinsurance OR micro- insurance OR “micro insurance” OR “microgroup lending” OR microfranchis* OR micro- franchis* OR “micro franchis*“ OR “micro credit*“ OR microcredit* OR micro-credit* OR saving* OR micro-saving* OR microsaving* OR “Smallholder financ*“ OR “rural financ*“ OR “rural credit”)) OR SU ( (micro-financ* OR “micro financ*“ OR microfinanc* OR micro-loan* OR microloan* OR “micro loan*“ OR microleas* OR micro-leas* OR “micro leas*“ OR microlending OR micro-lending OR “micro lending” OR microinsurance OR micro-insurance OR “micro insurance” OR “microgroup lending” OR microfranchis* OR micro-franchis* OR “micro franchis*“ OR “micro credit*“ OR microcredit* OR micro- credit* OR saving* OR micro-saving* OR microsaving* OR “Smallholder financ*“ OR “rural financ*“ OR “rural credit”))

TI ( (micro-financ* OR “micro financ*“ OR microfinanc* OR micro-loan* OR microloan* OR “micro loan*“ OR microleas* OR micro-leas* OR “micro leas*“ OR microlending OR micro-lending OR “micro lending” OR microinsurance OR micro-insurance OR “micro insurance” OR “microgroup lending” OR microfranchis* OR micro-franchis* OR “micro franchis*“ OR “micro credit*“ OR microcredit* OR micro-credit* OR saving* OR micro- saving* OR microsaving* OR “Smallholder financ*“ OR “rural financ*“ OR “rural credit” OR ROSCAs OR SHGs OR “group lending” OR “community saving*“ or “small loan*“ or “small lend*“ or ((bank or credit*) N3 cooperat*) or ((credit or loan* or lend*) N3 program*) or (community N3 (bank* or saving* or loan* or lend*)) or “income generat*“ or grameen OR ROSCA* OR stokvel* OR ((financial OR economic) N2 (literacy OR education OR skills OR training OR knowledge OR capab*)) OR banking OR budgeting OR “money manag*“ OR “consumption smoothing” OR rationing OR earmarking OR “bank account*“ OR “youth account*“ OR “lock box*“ OR “piggy bank*“ OR “saving box*“ OR ((access* OR participat*) N3 ( financ* OR credit OR saving* OR loan* OR lending)) OR “financial inclusion” OR “inclusive finance*“ OR fintech OR “mobile monies” OR M- PESA OR “mobile banking” OR cashless)) OR AB ( (micro-financ* OR “micro financ*“ OR microfinanc* OR micro-loan* OR microloan* OR “micro loan*“ OR microleas* OR micro-leas* OR “micro leas*“ OR microlending OR micro-lending OR “micro lending” OR microinsurance OR micro-insurance OR “micro insurance” OR “microgroup lending” OR microfranchis* OR micro-franchis* OR “micro franchis*“ OR “micro credit*“ OR microcredit* OR micro-credit* OR saving* OR micro-saving* OR microsaving* OR “Smallholder financ*“ OR “rural financ*“ OR “rural credit” OR ROSCAs OR SHGs OR “group lending” OR “community saving*“ or “small loan*“ or “small lend*“ or ((bank or credit*) N3 cooperat*) or ((credit or loan* or lend*) N3 program*) or (community N3 (bank* or saving* or loan* or lend*)) or “income generat*“ or grameen OR ROSCA* OR stokvel* OR ((financial OR economic) N2 (literacy OR education OR skills OR training OR knowledge OR capab*)) OR banking OR budgeting OR “money manag*“ OR “consumption smoothing” OR rationing OR earmarking OR “bank account*“ OR “youth account*“ OR “lock box*“ OR “piggy bank*“ OR “saving box*“ OR ((access* OR participat*) N3 ( financ* OR credit OR saving* OR loan* OR lending)) OR “financial inclusion” OR “inclusive finance*“ OR fintech OR “mobile monies” OR M-PESA OR “mobile banking” OR cashless)) OR SU ( (micro-financ* OR “micro financ*“ OR microfinanc* OR micro-loan* OR microloan* OR “micro loan*“ OR microleas* OR micro- leas* OR “micro leas*“ OR microlending OR micro-lending OR “micro lending” OR microinsurance OR micro-insurance OR “micro insurance” OR “microgroup lending” OR microfranchis* OR micro-franchis* OR “micro franchis*“ OR “micro credit*“ OR microcredit* OR micro-credit* OR saving* OR micro-saving* OR microsaving* OR “Smallholder financ*“ OR “rural financ*“ OR “rural credit” OR ROSCAs OR SHGs OR “group lending” OR “community saving*“ or “small loan*“ or “small lend*“ or ((bank or credit*) N3 cooperat*) or ((credit or loan* or lend*) N3 program*) or (community N3 (bank* or saving* or loan* or lend*)) or “income generat*“ or grameen OR ROSCA* OR stokvel* OR ((financial OR economic) N2 (literacy OR education OR skills OR training OR knowledge OR capab*)) OR banking OR budgeting OR “money manag*“ OR “consumption smoothing” OR rationing OR earmarking OR “bank account*“ OR “youth account*“ OR “lock box*“ OR “piggy bank*“ OR “saving box*“ OR ((access* OR participat*) N3 ( financ* OR credit OR saving* OR loan* OR lending)) OR “financial inclusion” OR “inclusive finance*“ OR fintech OR “mobile monies” OR M-PESA OR “mobile banking” OR cashless))

TI ( ((access* OR participat*) N3 ( financ* OR microfinanc* OR micro-financ* OR credit OR saving* OR loan* OR lending))) OR AB ( ((access* OR participat*) N3 ( financ* OR microfinanc* OR micro-financ* OR credit OR saving* OR loan* OR lending))) OR SU ( ((access* OR participat*) N3 ( financ* OR microfinanc* OR micro-financ* OR credit OR saving* OR loan* OR lending)))

Study design search terms

TI ( ((review N3 (effectiveness or effects or systemat* or synth* or integrat* or methodologic* or quantitative or evidence or literature or scoping) or “evidence gap” or “gap map”)) OR AB ( ((review N3 (effectiveness or effects or systemat* or synth* or integrat* or methodologic* or quantitative or evidence or literature or scoping) or “evidence gap” or “gap map”)) OR SU ( ((review N3 (effectiveness or effects or systemat* or synth* or integrat* or methodologic* or quantitative or evidence or literature or scoping) or “evidence gap” or “gap map”))

TI ( (“Meta regression” or “meta synth*“ or “meta-synth*“ or “meta analy*“ or “metaanaly*“ or “meta-analy*“ or “metanaly*“ or “Metaregression” or “Meta-regression” or “Methodologic* overview” or “pool* analys*“ or “pool* data” or “Quantitative* overview” or “research integration”)) OR AB ( (“Meta regression” or “meta synth*“ or “meta-synth*“ or “meta analy*“ or “metaanaly*“ or “meta-analy*“ or “metanaly*“ or “Metaregression” or “Meta-regression” or “Methodologic* overview” or “pool* analys*“ or “pool* data” or “Quantitative* overview” or “research integration”)) OR SU ( (“Meta regression” or “meta synth*“ or “meta-synth*“ or “meta analy*“ or “metaanaly*“ or “meta-analy*“ or “metanaly*“ or “Metaregression” or “Meta-regression” or “Methodologic* overview” or “pool* analys*“ or “pool* data” or “Quantitative* overview” or “research integration”))

TI ( (“quasi experiment*“ or quasi-experiment* or “random* control* trial*“ or “random* trial*“ or rct* or (random* N3 allocat*) or evaluat* or impact* or assess* or dif-dif or psm or “double difference” or difference-in-difference or rdd or “difference in difference” or “statistical matching*“ or “propensity score matching” or “covariate matching” or “coarsened-exact matching” or “propensity-weighted” or “multiple regression” or “statistical regression” or “regression discontinuity*“ or “cohort analysis” or “quantitative method*“ or “program* evaluation” or “interrupted time series” or (before N5 after) or (pre N5 post) or ((pretest or “pre test”) and (posttest or “post test”)) or ((“fixed effect*“ or “random effect*“) N3 (model or estimation)) or “instrumental variable” or “synthetic control” or ((quantitative or qualitative or “comparison group*“ or counterfactual or “counter factual” or counter-factual or experiment* or panel or cross-sectional) N3 (design or study or analysis)))) OR AB ( (“quasi experiment*“ or quasi-experiment* or “random* control* trial*“ or “random* trial*“ or rct* or (random* N3 allocat*) or evaluat* or impact* or assess* or dif-dif or psm or “double difference” or difference-in-difference or rdd or “difference in difference” or “statistical matching*“ or “propensity score matching” or “covariate matching” or “coarsened-exact matching” or “propensity- weighted” or “multiple regression” or “statistical regression” or “regression discontinuity*“ or “cohort analysis” or “quantitative method*“ or “program* evaluation” or “interrupted time series” or (before N5 after) or (pre N5 post) or ((pretest or “pre test”) and (posttest or “post test”)) or ((“fixed effect*“ or “random effect*“) N3 (model or estimation)) or “instrumental variable” or “synthetic control” or ((quantitative or qualitative or “comparison group*“ or counterfactual or “counter factual” or counter- factual or experiment* or panel or cross-sectional) N3 (design or study or analysis)))) OR SU ( (“quasi experiment*“ or quasi-experiment* or “random* control* trial*“ or “random* trial*“ or rct* or (random* N3 allocat*) or evaluat* or impact* or assess* or dif- dif or psm or “double difference” or difference-in-difference or rdd or “difference in difference” or “statistical matching*“ or “propensity score matching” or “covariate matching” or “coarsened-exact matching” or “propensity-weighted” or “multiple regression” or “statistical regression” or “regression discontinuity*“ or “cohort analysis” or “quantitative method*“ or “program* evaluation” or “interrupted time series” or (before N5 after) or (pre N5 post) or ((pretest or “pre test”) and (posttest or “post test”)) or ((“fixed effect*“ or “random effect*“) N3 (model or estimation)) or “instrumental variable” or “synthetic control” or ((quantitative or qualitative or “comparison group*“ or counterfactual or “counter factual” or counter-factual or experiment* or panel or cross- sectional) N3 (design or study or analysis))))

TI ( ((review N3 (effectiveness or effects or systemat* or synth* or integrat* or methodologic* or quantitative or evidence or literature or scoping) or “evidence gap” or “gap map”)) OR AB ( ((review N3 (effectiveness or effects or systemat* or synth* or integrat* or methodologic* or quantitative or evidence or literature or scoping) or “evidence gap” or “gap map”)) OR SU ( ((review N3 (effectiveness or effects or systemat* or synth* or integrat* or methodologic* or quantitative or evidence or literature or scoping) or “evidence gap” or “gap map”))

TI ( (“Meta regression” or “meta synth*“ or “meta-synth*“ or “meta analy*“ or “metaanaly*“ or “meta-analy*“ or “metanaly*“ or “Metaregression” or “Meta-regression” or “Methodologic* overview” or “pool* analys*“ or “pool* data” or “Quantitative* overview” or “research integration”)) OR AB ( (“Meta regression” or “meta synth*“ or “meta-synth*“ or “meta analy*“ or “metaanaly*“ or “meta-analy*“ or “metanaly*“ or “Metaregression” or “Meta-regression” or “Methodologic* overview” or “pool* analys*“ or “pool* data” or “Quantitative* overview” or “research integration”)) OR SU ( (“Meta regression” or “meta synth*“ or “meta-synth*“ or “meta analy*“ or “metaanaly*“ or

“meta-analy*“ or “metanaly*“ or “Metaregression” or “Meta-regression” or “Methodologic* overview” or “pool* analys*“ or “pool* data” or “Quantitative* overview” or “research integration”))

TI ( (“quasi experiment*“ or quasi-experiment* or “random* control* trial*“ or “random* trial*“ or rct* or (random* N3 allocat*) or evaluat* or impact* or assess* or dif-dif or psm or “double difference” or difference-in-difference or rdd or “difference in difference” or “statistical matching*“ or “propensity score matching” or “covariate matching” or “coarsened-exact matching” or “propensity-weighted” or “multiple regression” or “statistical regression” or “regression discontinuity*“ or “cohort analysis” or “quantitative method*“ or “program* evaluation” or “interrupted time series” or (before N5 after) or (pre N5 post) or ((pretest or “pre test”) and (posttest or “post test”)) or ((“fixed effect*“ or “random effect*“) N3 (model or estimation)) or “instrumental variable” or “synthetic control” or ((quantitative or qualitative or “comparison group*“ or counterfactual or “counter factual” or counter-factual or experiment* or panel or cross-sectional) N3 (design or study or analysis)))) OR AB ( (“quasi experiment*“ or quasi-experiment* or “random* control* trial*“ or “random* trial*“ or rct* or (random* N3 allocat*) or evaluat* or impact* or assess* or dif-dif or psm or “double difference” or difference-in-difference or rdd or “difference in difference” or “statistical matching*“ or “propensity score matching” or “covariate matching” or “coarsened-exact matching” or “propensity- weighted” or “multiple regression” or “statistical regression” or “regression discontinuity*“ or “cohort analysis” or “quantitative method*“ or “program* evaluation” or “interrupted time series” or (before N5 after) or (pre N5 post) or ((pretest or “pre test”) and (posttest or “post test”)) or ((“fixed effect*“ or “random effect*“) N3 (model or estimation)) or “instrumental variable” or “synthetic control” or ((quantitative or qualitative or “comparison group*“ or counterfactual or “counter factual” or counter- factual or experiment* or panel or cross-sectional) N3 (design or study or analysis)))) OR SU ( (“quasi experiment*“ or quasi-experiment* or “random* control* trial*“ or “random* trial*“ or rct* or (random* N3 allocat*) or evaluat* or impact* or assess* or dif- dif or psm or “double difference” or difference-in-difference or rdd or “difference in difference” or “statistical matching*“ or “propensity score matching” or “covariate matching” or “coarsened-exact matching” or “propensity-weighted” or “multiple regression” or “statistical regression” or “regression discontinuity*“ or “cohort analysis” or “quantitative method*“ or “program* evaluation” or “interrupted time series” or (before N5 after) or (pre N5 post) or ((pretest or “pre test”) and (posttest or “post test”)) or ((“fixed effect*“ or “random effect*“) N3 (model or estimation)) or “instrumental variable” or “synthetic control” or ((quantitative or qualitative or “comparison group*“ or counterfactual or “counter factual” or counter-factual or experiment* or panel or cross- sectional) N3 (design or study or analysis))))

MSME search terms

TI ( (msme or msmes or sme or smes or ((micro or small or medium) N2 (enterprise* or business* or firm or firms)) or OR “micro enterprise*“ OR microenterprise* OR micro- enterprise* or household* or “small holder*“ or smallholder* or farmer* or ((“single person*“ N3 (enterprise* or business*)) or ((communit* or women*) N2 group*))) OR AB ( (msme or msmes or sme or smes or ((micro or small or medium) N2 (enterprise* or business* or firm or firms)) or OR “micro enterprise*“ OR microenterprise* OR micro- enterprise* or household* or “small holder*“ or smallholder* or farmer* or ((“single person*“ N3 (enterprise* or business*)) or ((communit* or women*) N2 group*))) AND SU ( (msme or msmes or sme or smes or ((micro or small or medium) N2 (enterprise* or business* or firm or firms)) or OR “micro enterprise*“ OR microenterprise* OR micro- enterprise* or household* or “small holder*“ or smallholder* or farmer* or ((“single person*“ N3 (enterprise* or business*)) or ((communit* or women*) N2 group*)))

TI ( (msme or msmes or sme or smes or ((micro or small or medium) N2 (enterprise* or business*)) or OR “micro enterprise*“ OR microenterprise* OR micro-enterprise* or household* or “small holder*“ or smallholder* or farmer* or ((“single person*“ N3 (enterprise* or business*)) or ((communit* or women*) N2 group*))) OR AB ( (msme or msmes or sme or smes or ((micro or small or medium) N2 (enterprise* or business*)) or OR “micro enterprise*“ OR microenterprise* OR micro-enterprise* or household* or “small holder*“ or smallholder* or farmer* or ((“single person*“ N3 (enterprise* or business*)) or ((communit* or women*) N2 group*))) AND SU ( (msme or msmes or sme or smes or ((micro or small or medium) N2 (enterprise* or business*)) or OR “micro enterprise*“ OR microenterprise* OR micro-enterprise* or household* or “small holder*“ or smallholder* or farmer* or ((“single person*“ N3 (enterprise* or business*)) or ((communit* or women*) N2 group*)))

LMIC search terms

TI ( (afghanistan or albania or algeria or “american samoa” or angola or “antigua and barbuda” or antigua or barbuda or argentina or armenia or armenian or aruba or azerbaijan or bahrain or bangladesh or barbados or republic of belarus or belarus or byelarus or belorussia or byelorussian or belize or “british honduras” or benin or dahomey or bhutan or bolivia or “bosnia and herzegovina” or bosnia or herzegovina or botswana or bechuanaland or brazil or brasil or bulgaria or “burkina faso” or “burkina fasso” or “upper volta” or burundi or urundi or “cabo verde” or “cape verde” or cambodia or kampuchea or “khmer republic” or cameroon or cameron or cameroun or “central african republic” or “ubangi shari” or chad or chile or china or colombia or comoros or “comoro islands” or “iles comores” or mayotte or “democratic republic of the congo” or “democratic republic congo” or congo or zaire or “costa rica” or “cote divoire” or “cote d ivoire” or “cote divoire” or “cote d ivoire” or “ivory coast” or croatia or cuba or cyprus or “czech republic” or czechoslovakia or djibouti or “french somaliland” or dominica or “dominican republic” or ecuador or egypt or “united arab republic” or “el salvador” or “equatorial guinea” or “spanish guinea” or eritrea or estonia or eswatini or swaziland or ethiopia or fiji or gabon or “gabonese republic” or gambia or “georgia (republic) “ or georgian or ghana or “gold coast” or gibraltar or greece or grenada or guam or guatemala or guinea or “guinea bissau” or guyana or “british guiana” or haiti or hispaniola or honduras or hungary or india or indonesia or timor or iran or iraq or “isle of man” or jamaica or jordan or kazakhstan or kazakh or kenya or “democratic peoples republic of korea” or “republic of korea” or “north korea” or “south korea” or korea or kosovo or kyrgyzstan or kirghizia or kirgizstan or “kyrgyz republic” or kirghiz or laos or “lao pdr” or “lao people’s democratic republic” or latvia or lebanon or “lebanese republic” or lesotho or basutoland or liberia or libya or “libyan arab jamahiriya” or lithuania or macau or macao or “republic of north macedonia” or macedonia or madagascar or “malagasy republic” or malawi or nyasaland or malaysia or “malay federation” or “malaya federation” or maldives or “indian ocean islands” or “indian ocean” or mali or malta or micronesia or “federated states of micronesia” or kiribati or “marshall islands” or nauru or “northern mariana islands” or palau or tuvalu or mauritania or mauritius or mexico or moldova or moldovian or mongolia or montenegro or morocco or ifni or mozambique or “portuguese east africa” or myanmar or burma or namibia or nepal or “netherlands antilles” or nicaragua or niger or nigeria or oman or muscat or pakistan or panama or “papua new guinea” or “new guinea” or paraguay or peru or philippines or philipines or phillipines or phillippines or poland or “polish people’s republic” or portugal or “portuguese republic” or “puerto rico” or romania or russia or “russian federation” or ussr or “soviet union or union of soviet socialist republics” or rwanda or ruanda or samoa or “pacific islands” or polynesia or “samoan islands” or “navigator island” or “navigator islands” or “sao tome and principe” or “saudi arabia” or senegal or serbia or seychelles or “sierra leone” or slovakia or “slovak republic” or slovenia or melanesia or “solomon island” or “solomon islands” or “norfolk island” or “norfolk islands” or somalia or “south africa” or “south sudan” or “sri lanka” or ceylon or “saint kitts and nevis” or “st. kitts and nevis” or “saint lucia” or “st. lucia” or “saint vincent and the grenadines” or “saint vincent” or “st. vincent” or grenadines or sudan or suriname or surinam or “dutch guiana” or “netherlands guiana” or syria or “syrian arab republic” or tajikistan or tadjikistan or tadzhikistan or tadzhik or tanzania or tanganyika or thailand or siam or “timor leste” or “east timor” or togo or “togolese republic” or tonga or “trinidad and tobago” or trinidad or tobago or tunisia or turkey or turkmenistan or turkmen or uganda or ukraine or uruguay or uzbekistan or uzbek or vanuatu or “new hebrides” or venezuela or vietnam or “viet nam” or “middle east” or “west bank” or gaza or palestine or yemen or yugoslavia or zambia or zimbabwe or “northern rhodesia” or “global south” or “africa south of the sahara” or “sub-saharan africa” or “subsaharan africa” or “africa, central” or “central africa” or “africa, northern” or “north africa” or “northern africa” or magreb or maghrib or sahara or “africa, southern” or “southern africa” or “africa, eastern” or “east africa” or “eastern africa” or “africa, western” or “west africa” or “western africa” or “west indies” or “indian ocean islands” or caribbean or “central america” or “latin america” or “south and central america” or “south america” or “asia, central” or “central asia” or “asia, northern” or “north asia” or “northern asia” or “asia, southeastern” or “southeastern asia” or “south eastern asia” or “southeast asia” or “south east asia” or “asia, western” or “western asia” or “europe, eastern” or “east europe” or “eastern europe” or “developing country” or “developing countries” or “developing nation*“ or “developing population*“ or “developing world” or “less developed countr*“ or “less developed nation*“ or “less developed population*“ or “less developed world” or “lesser developed countr*“ or “lesser developed nation*“ or “lesser developed population*“ or “lesser developed world” or “under developed countr*“ or “under developed nation*“ or “under developed population*“ or “under developed world” or “underdeveloped countr*“ or “underdeveloped nation*“ or “underdeveloped population*“ or “underdeveloped world” or “middle income countr*“ or “middle income nation*“ or “middle income population*“ or “low income countr*“ or “low income nation*“ or “low income population*“ or “lower income countr*“ or “lower income nation*“ or “lower income population*“ or “underserved countr*“ or “underserved nation*“ or “underserved population*“ or “underserved world” or “under served countr*“ or “under served nation*“ or “under served population*“ or “under served world” or “deprived countr*“ or “deprived nation*“ or “deprived population*“ or “deprived world” or “poor countr*“ or “poor nation*“ or “poor population*“ or “poor world” or “poorer countr*“ or “poorer nation*“ or “poorer population*“ or “poorer world” or “developing econom*“ or “less developed econom*“ or “lesser developed econom*“ or “under developed econom*“ or “underdeveloped econom*“ or “middle income econom*“ or “low income econom*“ or “lower income econom*“ or “low gdp” or “low gnp” or “low gross domestic” or “low gross national” or “lower gdp” or “lower gnp” or “lower gross domestic” or “lower gross national” or lmic or lmics or “third world” or “lami countr*“ or “transitional countr*“ or “emerging economies” or “emerging nation*“)) OR AB ( (afghanistan or albania or algeria or “american samoa” or angola or “antigua and barbuda” or antigua or barbuda or argentina or armenia or armenian or aruba or azerbaijan or bahrain or bangladesh or barbados or republic of belarus or belarus or byelarus or belorussia or byelorussian or belize or “british honduras” or benin or dahomey or bhutan or bolivia or “bosnia and herzegovina” or bosnia or herzegovina or botswana or bechuanaland or brazil or brasil or bulgaria or “burkina faso” or “burkina fasso” or “upper volta” or burundi or urundi or “cabo verde” or “cape verde” or cambodia or kampuchea or “khmer republic” or cameroon or cameron or cameroun or “central african republic” or “ubangi shari” or chad or chile or china or colombia or comoros or “comoro islands” or “iles comores” or mayotte or “democratic republic of the congo” or “democratic republic congo” or congo or zaire or “costa rica” or “cote divoire” or “cote d ivoire” or “cote divoire” or “cote d ivoire” or “ivory coast” or croatia or cuba or cyprus or “czech republic” or czechoslovakia or djibouti or “french somaliland” or dominica or “dominican republic” or ecuador or egypt or “united arab republic” or “el salvador” or “equatorial guinea” or “spanish guinea” or eritrea or estonia or eswatini or swaziland or ethiopia or fiji or gabon or “gabonese republic” or gambia or “georgia (republic) “ or georgian or ghana or “gold coast” or gibraltar or greece or grenada or guam or guatemala or guinea or “guinea bissau” or guyana or “british guiana” or haiti or hispaniola or honduras or hungary or india or indonesia or timor or iran or iraq or “isle of man” or jamaica or jordan or kazakhstan or kazakh or kenya or “democratic peoples republic of korea” or “republic of korea” or “north korea” or “south korea” or korea or kosovo or kyrgyzstan or kirghizia or kirgizstan or “kyrgyz republic” or kirghiz or laos or “lao pdr” or “lao people’s democratic republic” or latvia or lebanon or “lebanese republic” or lesotho or basutoland or liberia or libya or “libyan arab jamahiriya” or lithuania or macau or macao or “republic of north macedonia” or macedonia or madagascar or “malagasy republic” or malawi or nyasaland or malaysia or “malay federation” or “malaya federation” or maldives or “indian ocean islands” or “indian ocean” or mali or malta or micronesia or “federated states of micronesia” or kiribati or “marshall islands” or nauru or “northern mariana islands” or palau or tuvalu or mauritania or mauritius or mexico or moldova or moldovian or mongolia or montenegro or morocco or ifni or mozambique or “portuguese east africa” or myanmar or burma or namibia or nepal or “netherlands antilles” or nicaragua or niger or nigeria or oman or muscat or pakistan or panama or “papua new guinea” or “new guinea” or paraguay or peru or philippines or philipines or phillipines or phillippines or poland or “polish people’s republic” or portugal or “portuguese republic” or “puerto rico” or romania or russia or “russian federation” or ussr or “soviet union or union of soviet socialist republics” or rwanda or ruanda or samoa or “pacific islands” or polynesia or “samoan islands” or “navigator island” or “navigator islands” or “sao tome and principe” or “saudi arabia” or senegal or serbia or seychelles or “sierra leone” or slovakia or “slovak republic” or slovenia or melanesia or “solomon island” or “solomon islands” or “norfolk island” or “norfolk islands” or somalia or “south africa” or “south sudan” or “sri lanka” or ceylon or “saint kitts and nevis” or “st. kitts and nevis” or “saint lucia” or “st. lucia” or “saint vincent and the grenadines” or “saint vincent” or “st. vincent” or grenadines or sudan or suriname or surinam or “dutch guiana” or “netherlands guiana” or syria or “syrian arab republic” or tajikistan or tadjikistan or tadzhikistan or tadzhik or tanzania or tanganyika or thailand or siam or “timor leste” or “east timor” or togo or “togolese republic” or tonga or “trinidad and tobago” or trinidad or tobago or tunisia or turkey or turkmenistan or turkmen or uganda or ukraine or uruguay or uzbekistan or uzbek or vanuatu or “new hebrides” or venezuela or vietnam or “viet nam” or “middle east” or “west bank” or gaza or palestine or yemen or yugoslavia or zambia or zimbabwe or “northern rhodesia” or “global south” or “africa south of the sahara” or “sub-saharan africa” or “subsaharan africa” or “africa, central” or “central africa” or “africa, northern” or “north africa” or “northern africa” or magreb or maghrib or sahara or “africa, southern” or “southern africa” or “africa, eastern” or “east africa” or “eastern africa” or “africa, western” or “west africa” or “western africa” or “west indies” or “indian ocean islands” or caribbean or “central america” or “latin america” or “south and central america” or “south america” or “asia, central” or “central asia” or “asia, northern” or “north asia” or “northern asia” or “asia, southeastern” or “southeastern asia” or “south eastern asia” or “southeast asia” or “south east asia” or “asia, western” or “western asia” or “europe, eastern” or “east europe” or “eastern europe” or “developing country” or “developing countries” or “developing nation*“ or “developing population*“ or “developing world” or “less developed countr*“ or “less developed nation*“ or “less developed population*“ or “less developed world” or “lesser developed countr*“ or “lesser developed nation*“ or “lesser developed population*“ or “lesser developed world” or “under developed countr*“ or “under developed nation*“ or “under developed population*“ or “under developed world” or “underdeveloped countr*“ or “underdeveloped nation*“ or “underdeveloped population*“ or “underdeveloped world” or “middle income countr*“ or “middle income nation*“ or “middle income population*“ or “low income countr*“ or “low income nation*“ or “low income population*“ or “lower income countr*“ or “lower income nation*“ or “lower income population*“ or “underserved countr*“ or “underserved nation*“ or “underserved population*“ or “underserved world” or “under served countr*“ or “under served nation*“ or “under served population*“ or “under served world” or “deprived countr*“ or “deprived nation*“ or “deprived population*“ or “deprived world” or “poor countr*“ or “poor nation*“ or “poor population*“ or “poor world” or “poorer countr*“ or “poorer nation*“ or “poorer population*“ or “poorer world” or “developing econom*“ or “less developed econom*“ or “lesser developed econom*“ or “under developed econom*“ or “underdeveloped econom*“ or “middle income econom*“ or “low income econom*“ or “lower income econom*“ or “low gdp” or “low gnp” or “low gross domestic” or “low gross national” or “lower gdp” or “lower gnp” or “lower gross domestic” or “lower gross national” or lmic or lmics or “third world” or “lami countr*“ or “transitional countr*“ or “emerging economies” or “emerging nation*“)) OR SU ( (afghanistan or albania or algeria or “american samoa” or angola or “antigua and barbuda” or antigua or barbuda or argentina or armenia or armenian or aruba or azerbaijan or bahrain or bangladesh or barbados or republic of belarus or belarus or byelarus or belorussia or byelorussian or belize or “british honduras” or benin or dahomey or bhutan or bolivia or “bosnia and herzegovina” or bosnia or herzegovina or botswana or bechuanaland or brazil or brasil or bulgaria or “burkina faso” or “burkina fasso” or “upper volta” or burundi or urundi or “cabo verde” or “cape verde” or cambodia or kampuchea or “khmer republic” or cameroon or cameron or cameroun or “central african republic” or “ubangi shari” or chad or chile or china or colombia or comoros or “comoro islands” or “iles comores” or mayotte or “democratic republic of the congo” or “democratic republic congo” or congo or zaire or “costa rica” or “cote divoire” or “cote d ivoire” or “cote divoire” or “cote d ivoire” or “ivory coast” or croatia or cuba or cyprus or “czech republic” or czechoslovakia or djibouti or “french somaliland” or dominica or “dominican republic” or ecuador or egypt or “united arab republic” or “el salvador” or “equatorial guinea” or “spanish guinea” or eritrea or estonia or eswatini or swaziland or ethiopia or fiji or gabon or “gabonese republic” or gambia or “georgia (republic) “ or georgian or ghana or “gold coast” or gibraltar or greece or grenada or guam or guatemala or guinea or “guinea bissau” or guyana or “british guiana” or haiti or hispaniola or honduras or hungary or india or indonesia or timor or iran or iraq or “isle of man” or jamaica or jordan or kazakhstan or kazakh or kenya or “democratic peoples republic of korea” or “republic of korea” or “north korea” or “south korea” or korea or kosovo or kyrgyzstan or kirghizia or kirgizstan or “kyrgyz republic” or kirghiz or laos or “lao pdr” or “lao people’s democratic republic” or latvia or lebanon or “lebanese republic” or lesotho or basutoland or liberia or libya or “libyan arab jamahiriya” or lithuania or macau or macao or “republic of north macedonia” or macedonia or madagascar or “malagasy republic” or malawi or nyasaland or malaysia or “malay federation” or “malaya federation” or maldives or “indian ocean islands” or “indian ocean” or mali or malta or micronesia or “federated states of micronesia” or kiribati or “marshall islands” or nauru or “northern mariana islands” or palau or tuvalu or mauritania or mauritius or mexico or moldova or moldovian or mongolia or montenegro or morocco or ifni or mozambique or “portuguese east africa” or myanmar or burma or namibia or nepal or “netherlands antilles” or nicaragua or niger or nigeria or oman or muscat or pakistan or panama or “papua new guinea” or “new guinea” or paraguay or peru or philippines or philipines or phillipines or phillippines or poland or “polish people’s republic” or portugal or “portuguese republic” or “puerto rico” or romania or russia or “russian federation” or ussr or “soviet union or union of soviet socialist republics” or rwanda or ruanda or samoa or “pacific islands” or polynesia or “samoan islands” or “navigator island” or “navigator islands” or “sao tome and principe” or “saudi arabia” or senegal or serbia or seychelles or “sierra leone” or slovakia or “slovak republic” or slovenia or melanesia or “solomon island” or “solomon islands” or “norfolk island” or “norfolk islands” or somalia or “south africa” or “south sudan” or “sri lanka” or ceylon or “saint kitts and nevis” or “st. kitts and nevis” or “saint lucia” or “st. lucia” or “saint vincent and the grenadines” or “saint vincent” or “st. vincent” or grenadines or sudan or suriname or surinam or “dutch guiana” or “netherlands guiana” or syria or “syrian arab republic” or tajikistan or tadjikistan or tadzhikistan or tadzhik or tanzania or tanganyika or thailand or siam or “timor leste” or “east timor” or togo or “togolese republic” or tonga or “trinidad and tobago” or trinidad or tobago or tunisia or turkey or turkmenistan or turkmen or uganda or ukraine or uruguay or uzbekistan or uzbek or vanuatu or “new hebrides” or venezuela or vietnam or “viet nam” or “middle east” or “west bank” or gaza or palestine or yemen or yugoslavia or zambia or zimbabwe or “northern rhodesia” or “global south” or “africa south of the sahara” or “sub-saharan africa” or “subsaharan africa” or “africa, central” or “central africa” or “africa, northern” or “north africa” or “northern africa” or magreb or maghrib or sahara or “africa, southern” or “southern africa” or “africa, eastern” or “east africa” or “eastern africa” or “africa, western” or “west africa” or “western africa” or “west indies” or “indian ocean islands” or caribbean or “central america” or “latin america” or “south and central america” or “south america” or “asia, central” or “central asia” or “asia, northern” or “north asia” or “northern asia” or “asia, southeastern” or “southeastern asia” or “south eastern asia” or “southeast asia” or “south east asia” or “asia, western” or “western asia” or “europe, eastern” or “east europe” or “eastern europe” or “developing country” or “developing countries” or “developing nation*“ or “developing population*“ or “developing world” or “less developed countr*“ or “less developed nation*“ or “less developed population*“ or “less developed world” or “lesser developed countr*“ or “lesser developed nation*“ or “lesser developed population*“ or “lesser developed world” or “under developed countr*“ or “under developed nation*“ or “under developed population*“ or “under developed world” or “underdeveloped countr*“ or “underdeveloped nation*“ or “underdeveloped population*“ or “underdeveloped world” or “middle income countr*“ or “middle income nation*“ or “middle income population*“ or “low income countr*“ or “low income nation*“ or “low income population*“ or “lower income countr*“ or “lower income nation*“ or “lower income population*“ or “underserved countr*“ or “underserved nation*“ or “underserved population*“ or “underserved world” or “under served countr*“ or “under served nation*“ or “under served population*“ or “under served world” or “deprived countr*“ or “deprived nation*“ or “deprived population*“ or “deprived world” or “poor countr*“ or “poor nation*“ or “poor population*“ or “poor world” or “poorer countr*“ or “poorer nation*“ or “poorer population*“ or “poorer world” or “developing econom*“ or “less developed econom*“ or “lesser developed econom*“ or “under developed econom*“ or “underdeveloped econom*“ or “middle income econom*“ or “low income econom*“ or “lower income econom*“ or “low gdp” or “low gnp” or “low gross domestic” or “low gross national” or “lower gdp” or “lower gnp” or “lower gross domestic” or “lower gross national” or lmic or lmics or “third world” or “lami countr*“ or “transitional countr*“ or “emerging economies” or “emerging nation*“))

TI ( (afghanistan or albania or algeria or “american samoa” or angola or “antigua and barbuda” or antigua or barbuda or argentina or armenia or armenian or aruba or azerbaijan or bahrain or bangladesh or barbados or republic of belarus or belarus or byelarus or belorussia or byelorussian or belize or “british honduras” or benin or dahomey or bhutan or bolivia or “bosnia and herzegovina” or bosnia or herzegovina or botswana or bechuanaland or brazil or brasil or bulgaria or “burkina faso” or “burkina fasso” or “upper volta” or burundi or urundi or “cabo verde” or “cape verde” or cambodia or kampuchea or “khmer republic” or cameroon or cameron or cameroun or “central african republic” or “ubangi shari” or chad or chile or china or colombia or comoros or “comoro islands” or “iles comores” or mayotte or “democratic republic of the congo” or “democratic republic congo” or congo or zaire or “costa rica” or “cote divoire” or “cote d ivoire” or “cote divoire” or “cote d ivoire” or “ivory coast” or croatia or cuba or cyprus or “czech republic” or czechoslovakia or djibouti or “french somaliland” or dominica or “dominican republic” or ecuador or egypt or “united arab republic” or “el salvador” or “equatorial guinea” or “spanish guinea” or eritrea or estonia or eswatini or swaziland or ethiopia or fiji or gabon or “gabonese republic” or gambia or “georgia (republic) “ or georgian or ghana or “gold coast” or gibraltar or greece or grenada or guam or guatemala or guinea or “guinea bissau” or guyana or “british guiana” or haiti or hispaniola or honduras or hungary or india or indonesia or timor or iran or iraq or “isle of man” or jamaica or jordan or kazakhstan or kazakh or kenya or “democratic peoples republic of korea” or “republic of korea” or “north korea” or “south korea” or korea or kosovo or kyrgyzstan or kirghizia or kirgizstan or “kyrgyz republic” or kirghiz or laos or “lao pdr” or “lao people’s democratic republic” or latvia or lebanon or “lebanese republic” or lesotho or basutoland or liberia or libya or “libyan arab jamahiriya” or lithuania or macau or macao or “republic of north macedonia” or macedonia or madagascar or “malagasy republic” or malawi or nyasaland or malaysia or “malay federation” or “malaya federation” or maldives or “indian ocean islands” or “indian ocean” or mali or malta or micronesia or “federated states of micronesia” or kiribati or “marshall islands” or nauru or “northern mariana islands” or palau or tuvalu or mauritania or mauritius or mexico or moldova or moldovian or mongolia or montenegro or morocco or ifni or mozambique or “portuguese east africa” or myanmar or burma or namibia or nepal or “netherlands antilles” or nicaragua or niger or nigeria or oman or muscat or pakistan or panama or “papua new guinea” or “new guinea” or paraguay or peru or philippines or philipines or phillipines or phillippines or poland or “polish people’s republic” or portugal or “portuguese republic” or “puerto rico” or romania or russia or “russian federation” or ussr or “soviet union or union of soviet socialist republics” or rwanda or ruanda or samoa or “pacific islands” or polynesia or “samoan islands” or “navigator island” or “navigator islands” or “sao tome and principe” or “saudi arabia” or senegal or serbia or seychelles or “sierra leone” or slovakia or “slovak republic” or slovenia or melanesia or “solomon island” or “solomon islands” or “norfolk island” or “norfolk islands” or somalia or “south africa” or “south sudan” or “sri lanka” or ceylon or “saint kitts and nevis” or “st. kitts and nevis” or “saint lucia” or “st. lucia” or “saint vincent and the grenadines” or “saint vincent” or “st. vincent” or grenadines or sudan or suriname or surinam or “dutch guiana” or “netherlands guiana” or syria or “syrian arab republic” or tajikistan or tadjikistan or tadzhikistan or tadzhik or tanzania or tanganyika or thailand or siam or “timor leste” or “east timor” or togo or “togolese republic” or tonga or “trinidad and tobago” or trinidad or tobago or tunisia or turkey or turkmenistan or turkmen or uganda or ukraine or uruguay or uzbekistan or uzbek or vanuatu or “new hebrides” or venezuela or vietnam or “viet nam” or “middle east” or “west bank” or gaza or palestine or yemen or yugoslavia or zambia or zimbabwe or “northern rhodesia” or “global south” or “africa south of the sahara” or “sub-saharan africa” or “subsaharan africa” or “africa, central” or “central africa” or “africa, northern” or “north africa” or “northern africa” or magreb or maghrib or sahara or “africa, southern” or “southern africa” or “africa, eastern” or “east africa” or “eastern africa” or “africa, western” or “west africa” or “western africa” or “west indies” or “indian ocean islands” or caribbean or “central america” or “latin america” or “south and central america” or “south america” or “asia, central” or “central asia” or “asia, northern” or “north asia” or “northern asia” or “asia, southeastern” or “southeastern asia” or “south eastern asia” or “southeast asia” or “south east asia” or “asia, western” or “western asia” or “europe, eastern” or “east europe” or “eastern europe” or “developing country” or “developing countries” or “developing nation*“ or “developing population*“ or “developing world” or “less developed countr*“ or “less developed nation*“ or “less developed population*“ or “less developed world” or “lesser developed countr*“ or “lesser developed nation*“ or “lesser developed population*“ or “lesser developed world” or “under developed countr*“ or “under developed nation*“ or “under developed population*“ or “under developed world” or “underdeveloped countr*“ or “underdeveloped nation*“ or “underdeveloped population*“ or “underdeveloped world” or “middle income countr*“ or “middle income nation*“ or “middle income population*“ or “low income countr*“ or “low income nation*“ or “low income population*“ or “lower income countr*“ or “lower income nation*“ or “lower income population*“ or “underserved countr*“ or “underserved nation*“ or “underserved population*“ or “underserved world” or “under served countr*“ or “under served nation*“ or “under served population*“ or “under served world” or “deprived countr*“ or “deprived nation*“ or “deprived population*“ or “deprived world” or “poor countr*“ or “poor nation*“ or “poor population*“ or “poor world” or “poorer countr*“ or “poorer nation*“ or “poorer population*“ or “poorer world” or “developing econom*“ or “less developed econom*“ or “lesser developed econom*“ or “under developed econom*“ or “underdeveloped econom*“ or “middle income econom*“ or “low income econom*“ or “lower income econom*“ or “low gdp” or “low gnp” or “low gross domestic” or “low gross national” or “lower gdp” or “lower gnp” or “lower gross domestic” or “lower gross national” or lmic or lmics or “third world” or “lami countr*“ or “transitional countr*“ or “emerging economies” or “emerging nation*“)) OR AB ( (afghanistan or albania or algeria or “american samoa” or angola or “antigua and barbuda” or antigua or barbuda or argentina or armenia or armenian or aruba or azerbaijan or bahrain or bangladesh or barbados or republic of belarus or belarus or byelarus or belorussia or byelorussian or belize or “british honduras” or benin or dahomey or bhutan or bolivia or “bosnia and herzegovina” or bosnia or herzegovina or botswana or bechuanaland or brazil or brasil or bulgaria or “burkina faso” or “burkina fasso” or “upper volta” or burundi or urundi or “cabo verde” or “cape verde” or cambodia or kampuchea or “khmer republic” or cameroon or cameron or cameroun or “central african republic” or “ubangi shari” or chad or chile or china or colombia or comoros or “comoro islands” or “iles comores” or mayotte or “democratic republic of the congo” or “democratic republic congo” or congo or zaire or “costa rica” or “cote divoire” or “cote d ivoire” or “cote divoire” or “cote d ivoire” or “ivory coast” or croatia or cuba or cyprus or “czech republic” or czechoslovakia or djibouti or “french somaliland” or dominica or “dominican republic” or ecuador or egypt or “united arab republic” or “el salvador” or “equatorial guinea” or “spanish guinea” or eritrea or estonia or eswatini or swaziland or ethiopia or fiji or gabon or “gabonese republic” or gambia or “georgia (republic) “ or georgian or ghana or “gold coast” or gibraltar or greece or grenada or guam or guatemala or guinea or “guinea bissau” or guyana or “british guiana” or haiti or hispaniola or honduras or hungary or india or indonesia or timor or iran or iraq or “isle of man” or jamaica or jordan or kazakhstan or kazakh or kenya or “democratic peoples republic of korea” or “republic of korea” or “north korea” or “south korea” or korea or kosovo or kyrgyzstan or kirghizia or kirgizstan or “kyrgyz republic” or kirghiz or laos or “lao pdr” or “lao people’s democratic republic” or latvia or lebanon or “lebanese republic” or lesotho or basutoland or liberia or libya or “libyan arab jamahiriya” or lithuania or macau or macao or “republic of north macedonia” or macedonia or madagascar or “malagasy republic” or malawi or nyasaland or malaysia or “malay federation” or “malaya federation” or maldives or “indian ocean islands” or “indian ocean” or mali or malta or micronesia or “federated states of micronesia” or kiribati or “marshall islands” or nauru or “northern mariana islands” or palau or tuvalu or mauritania or mauritius or mexico or moldova or moldovian or mongolia or montenegro or morocco or ifni or mozambique or “portuguese east africa” or myanmar or burma or namibia or nepal or “netherlands antilles” or nicaragua or niger or nigeria or oman or muscat or pakistan or panama or “papua new guinea” or “new guinea” or paraguay or peru or philippines or philipines or phillipines or phillippines or poland or “polish people’s republic” or portugal or “portuguese republic” or “puerto rico” or romania or russia or “russian federation” or ussr or “soviet union or union of soviet socialist republics” or rwanda or ruanda or samoa or “pacific islands” or polynesia or “samoan islands” or “navigator island” or “navigator islands” or “sao tome and principe” or “saudi arabia” or senegal or serbia or seychelles or “sierra leone” or slovakia or “slovak republic” or slovenia or melanesia or “solomon island” or “solomon islands” or “norfolk island” or “norfolk islands” or somalia or “south africa” or “south sudan” or “sri lanka” or ceylon or “saint kitts and nevis” or “st. kitts and nevis” or “saint lucia” or “st. lucia” or “saint vincent and the grenadines” or “saint vincent” or “st. vincent” or grenadines or sudan or suriname or surinam or “dutch guiana” or “netherlands guiana” or syria or “syrian arab republic” or tajikistan or tadjikistan or tadzhikistan or tadzhik or tanzania or tanganyika or thailand or siam or “timor leste” or “east timor” or togo or “togolese republic” or tonga or “trinidad and tobago” or trinidad or tobago or tunisia or turkey or turkmenistan or turkmen or uganda or ukraine or uruguay or uzbekistan or uzbek or vanuatu or “new hebrides” or venezuela or vietnam or “viet nam” or “middle east” or “west bank” or gaza or palestine or yemen or yugoslavia or zambia or zimbabwe or “northern rhodesia” or “global south” or “africa south of the sahara” or “sub-saharan africa” or “sub-saharan africa” or “africa, central” or “central africa” or “africa, northern” or “north africa” or “northern africa” or magreb or maghrib or sahara or “africa, southern” or “southern africa” or “africa, eastern” or “east africa” or “eastern africa” or “africa, western” or “west africa” or “western africa” or “west indies” or “indian ocean islands” or caribbean or “central america” or “latin america” or “south and central america” or “south america” or “asia, central” or “central asia” or “asia, northern” or “north asia” or “northern asia” or “asia, southeastern” or “southeastern asia” or “south eastern asia” or “southeast asia” or “south east asia” or “asia, western” or “western asia” or “europe, eastern” or “east europe” or “eastern europe” or “developing country” or “developing countries” or “developing nation*“ or “developing population*“ or “developing world” or “less developed countr*“ or “less developed nation*“ or “less developed population*“ or “less developed world” or “lesser developed countr*“ or “lesser developed nation*“ or “lesser developed population*“ or “lesser developed world” or “under developed countr*“ or “under developed nation*“ or “under developed population*“ or “under developed world” or “underdeveloped countr*“ or “underdeveloped nation*“ or “underdeveloped population*“ or “underdeveloped world” or “middle income countr*“ or “middle income nation*“ or “middle income population*“ or “low income countr*“ or “low income nation*“ or “low income population*“ or “lower income countr*“ or “lower income nation*“ or “lower income population*“ or “underserved countr*“ or “underserved nation*“ or “underserved population*“ or “underserved world” or “under served countr*“ or “under served nation*“ or “under served population*“ or “under served world” or “deprived countr*“ or “deprived nation*“ or “deprived population*“ or “deprived world” or “poor countr*“ or “poor nation*“ or “poor population*“ or “poor world” or “poorer countr*“ or “poorer nation*“ or “poorer population*“ or “poorer world” or “developing econom*“ or “less developed econom*“ or “lesser developed econom*“ or “under developed econom*“ or “underdeveloped econom*“ or “middle income econom*“ or “low income econom*“ or “lower income econom*“ or “low gdp” or “low gnp” or “low gross domestic” or “low gross national” or “lower gdp” or “lower gnp” or “lower gross domestic” or “lower gross national” or lmic or lmics or “third world” or “lami countr*“ or “transitional countr*“ or “emerging economies” or “emerging nation*“)) OR SU ( (afghanistan or albania or algeria or “american samoa” or angola or “antigua and barbuda” or antigua or barbuda or argentina or armenia or armenian or aruba or azerbaijan or bahrain or bangladesh or barbados or republic of belarus or belarus or byelarus or belorussia or byelorussian or belize or “british honduras” or benin or dahomey or bhutan or bolivia or “bosnia and herzegovina” or bosnia or herzegovina or botswana or bechuanaland or brazil or brasil or bulgaria or “burkina faso” or “burkina fasso” or “upper volta” or burundi or urundi or “cabo verde” or “cape verde” or cambodia or kampuchea or “khmer republic” or cameroon or cameron or cameroun or “central african republic” or “ubangi shari” or chad or chile or china or colombia or comoros or “comoro islands” or “iles comores” or mayotte or “democratic republic of the congo” or “democratic republic congo” or congo or zaire or “costa rica” or “cote divoire” or “cote d ivoire” or “cote divoire” or “cote d ivoire” or “ivory coast” or croatia or cuba or cyprus or “czech republic” or czechoslovakia or djibouti or “french somaliland” or dominica or “dominican republic” or ecuador or egypt or “united arab republic” or “el salvador” or “equatorial guinea” or “spanish guinea” or eritrea or estonia or eswatini or swaziland or ethiopia or fiji or gabon or “gabonese republic” or gambia or “georgia (republic) “ or georgian or ghana or “gold coast” or gibraltar or greece or grenada or guam or guatemala or guinea or “guinea bissau” or guyana or “british guiana” or haiti or hispaniola or honduras or hungary or india or indonesia or timor or iran or iraq or “isle of man” or jamaica or jordan or kazakhstan or kazakh or kenya or “democratic peoples republic of korea” or “republic of korea” or “north korea” or “south korea” or korea or kosovo or kyrgyzstan or kirghizia or kirgizstan or “kyrgyz republic” or kirghiz or laos or “lao pdr” or “lao people’s democratic republic” or latvia or lebanon or “lebanese republic” or lesotho or basutoland or liberia or libya or “libyan arab jamahiriya” or lithuania or macau or macao or “republic of north macedonia” or macedonia or madagascar or “malagasy republic” or malawi or nyasaland or malaysia or “malay federation” or “malaya federation” or maldives or “indian ocean islands” or “indian ocean” or mali or malta or micronesia or “federated states of micronesia” or kiribati or “marshall islands” or nauru or “northern mariana islands” or palau or tuvalu or mauritania or mauritius or mexico or moldova or moldovian or mongolia or montenegro or morocco or ifni or mozambique or “portuguese east africa” or myanmar or burma or namibia or nepal or “netherlands antilles” or nicaragua or niger or nigeria or oman or muscat or pakistan or panama or “papua new guinea” or “new guinea” or paraguay or peru or philippines or philipines or phillipines or phillippines or poland or “polish people’s republic” or portugal or “portuguese republic” or “puerto rico” or romania or russia or “russian federation” or ussr or “soviet union or union of soviet socialist republics” or rwanda or ruanda or samoa or “pacific islands” or polynesia or “samoan islands” or “navigator island” or “navigator islands” or “sao tome and principe” or “saudi arabia” or senegal or serbia or seychelles or “sierra leone” or slovakia or “slovak republic” or slovenia or melanesia or “solomon island” or “solomon islands” or “norfolk island” or “norfolk islands” or somalia or “south africa” or “south sudan” or “sri lanka” or ceylon or “saint kitts and nevis” or “st. kitts and nevis” or “saint lucia” or “st. lucia” or “saint vincent and the grenadines” or “saint vincent” or “st. vincent” or grenadines or sudan or suriname or surinam or “dutch guiana” or “netherlands guiana” or syria or “syrian arab republic” or tajikistan or tadjikistan or tadzhikistan or tadzhik or tanzania or tanganyika or thailand or siam or “timor leste” or “east timor” or togo or “togolese republic” or tonga or “trinidad and tobago” or trinidad or tobago or tunisia or turkey or turkmenistan or turkmen or uganda or ukraine or uruguay or uzbekistan or uzbek or vanuatu or “new hebrides” or venezuela or vietnam or “viet nam” or “middle east” or “west bank” or gaza or palestine or yemen or yugoslavia or zambia or zimbabwe or “northern rhodesia” or “global south” or “africa south of the sahara” or “sub-saharan africa” or “subsaharan africa” or “africa, central” or “central africa” or “africa, northern” or “north africa” or “northern africa” or magreb or maghrib or sahara or “africa, southern” or “southern africa” or “africa, eastern” or “east africa” or “eastern africa” or “africa, western” or “west africa” or “western africa” or “west indies” or “indian ocean islands” or caribbean or “central america” or “latin america” or “south and central america” or “south america” or “asia, central” or “central asia” or “asia, northern” or “north asia” or “northern asia” or “asia, southeastern” or “southeastern asia” or “south eastern asia” or “southeast asia” or “south east asia” or “asia, western” or “western asia” or “europe, eastern” or “east europe” or “eastern europe” or “developing country” or “developing countries” or “developing nation*“ or “developing population*“ or “developing world” or “less developed countr*“ or “less developed nation*“ or “less developed population*“ or “less developed world” or “lesser developed countr*“ or “lesser developed nation*“ or “lesser developed population*“ or “lesser developed world” or “under developed countr*“ or “under developed nation*“ or “under developed population*“ or “under developed world” or “underdeveloped countr*“ or “underdeveloped nation*“ or “underdeveloped population*“ or “underdeveloped world” or “middle income countr*“ or “middle income nation*“ or “middle income population*“ or “low income countr*“ or “low income nation*“ or “low income population*“ or “lower income countr*“ or “lower income nation*“ or “lower income population*“ or “underserved countr*“ or “underserved nation*“ or “underserved population*“ or “underserved world” or “under served countr*“ or “under served nation*“ or “under served population*“ or “under served world” or “deprived countr*“ or “deprived nation*“ or “deprived population*“ or “deprived world” or “poor countr*“ or “poor nation*“ or “poor population*“ or “poor world” or “poorer countr*“ or “poorer nation*“ or “poorer population*“ or “poorer world” or “developing econom*“ or “less developed econom*“ or “lesser developed econom*“ or “under developed econom*“ or “underdeveloped econom*“ or “middle income econom*“ or “low income econom*“ or “lower income econom*“ or “low gdp” or “low gnp” or “low gross domestic” or “low gross national” or “lower gdp” or “lower gnp” or “lower gross domestic” or “lower gross national” or lmic or lmics or “third world” or “lami countr*“ or “transitional countr*“ or “emerging economies” or “emerging nation*“))

**Appendix C: Coding tool**

| Study design | - Experimental-RCT/natural experiment - Non-experimental/causal-comparative - Systematic Reviews |
| --- | --- |
| Publication status | - Completed - Ongoing |
| Population | - Rural/remote - Urban - Both rural and urban - Women (if majority - Youth - Older persons - Poor and disadvantaged - Humanitarian settings - People with disabilities - Unclear |
| Country by Region | - East Asia & Pacific - Latin America & Caribbean - Middle East & North Africa - North America - South Asia - Sub-Saharan Africa - Europe & Central Asia |
| Country by Income | - Low-income economies - Lower-middle-income economies - Upper-middle-income economies |
| Types of firm receiving the financial intervention | - Microenterprise (including single person/household) - Community group - Small enterprise - Medium enterprise |

| Firm sectors | - Agriculture - Construction - Manufacturing - Services - Transportation and communication - Wholesale and retail trade - Other services - Not reported |
| --- | --- |
| Scope/Geographical Coverage | - Local - Regional - National - Unclear |
